# Single-cell RNA sequencing reveals B cell-T cell interactions in vascular adventitia of hyperhomocysteinemia-accelerated atherosclerosis

**DOI:** 10.1007/s13238-021-00904-0

**Published:** 2022-02-17

**Authors:** Xiaolong Ma, Jiacheng Deng, Lulu Han, Yuwei Song, Yutong Miao, Xing Du, Guohui Dang, Dongmin Yang, Bitao Zhong, Changtao Jiang, Wei Kong, Qingbo Xu, Juan Feng, Xian Wang

**Affiliations:** 1 Department of Physiology and Pathophysiology, School of Basic Medical Sciences, Key Laboratory of Molecular Cardiovascular Science, Ministry of Education, Peking University, Beijing, 100191 China; 2 Cardiovascular Division, BHF Center of Vascular Regeneration, King’s College London, London, UK; 3 Department of Clinical Laboratory, Beijing Chao-Yang Hospital, Capital Medical University, Beijing, 100020 China; 4 Department of Cardiology, The First Affiliated Hospital, Zhejiang University, Hangzhou, 310003 China

**Dear Editor**

Cardiovascular disease (CVD) is the leading cause of death around the world (Truelsen, et al., 2015). Atherosclerosis, the dominant underlying cause of CVD, is a chronic inflammatory disease characterized by lipid accumulation and immune cell infiltration in plaques and vessels (Weber, et al., 1950). The immune microenvironment is critical for the development of atherosclerosis. Homocysteine (Hcy) is an intermediate product of methionine metabolism, and its elevation in plasma (>15 μmol/L), known as hyperhomocysteinemia (HHcy) , is an independent risk factor for atherosclerosis. HHcy is more common in Asia because of genetic factors and dietary habits (Huo, et al., 2015). folic acid supplement is one of the most important way to treat HHcy in clinic. Although HHcy potentiates atherosclerosis mainly through endothelial injury and inflammatory activation (Luo, et al., 2016), a comprehensive understanding of the immune microenvironment and potential mechanisms in HHcy-accelerated atherosclerotic aortas (HHcy-AA) is still lacking.

With the development and application of single-cell RNA sequencing (scRNA-seq), elucidation of complex cellular compositions and phenotypic heterogeneity of vascular immune cells has recently become possible. Firstly, we established a HHcy mouse model that has been proven to successfully induce inflammatory cell infiltration and aggravate atherosclerosis (Dai, et al., 2006). Female ApoE^−/−^ mice were fed a normal chow diet (CD) with drinking water supplemented with or without 1.8 g/L Hcy for 4 weeks. Western diet (WD)-fed ApoE^−/−^ mice were also included as an additional group. Single live CD45^+^ leukocytes were then sorted from isolated aortas (without adipose tissue) from three groups by fluorescence-activated cell sorting (FACS) and subjected to scRNA-seq (Fig. S1A and S1B). The concentration of Hcy in plasma was successfully increased by Hcy administration in the HHcy ApoE^−/−^ group (Fig. S1C). Oil Red O staining of aortic roots showed significantly increased plaque formation in HHcy ApoE^−/−^ mice and WD ApoE^−/−^ mice, confirming the aggravation of atherosclerosis by HHcy and WD (Fig. S1D). After a standard preprocessing workflow and exclusion of nonimmune cells with the R package Seurat (Stuart, et al., 2019), we obtained a total of 20,309 cells for further analysis (Fig. S1E–G).

Unsupervised graph-based clustering revealed 15 immune cell clusters (Fig. S2A and S2B), as shown in the t-distributed stochastic neighbor embedding (t-SNE) plot (Fig. 1A). All identified cell clusters were found in three groups and can be grouped as six immune cell types, including B cells (*Cd79a*, *C79b*, and *Cd19*), macrophages (*Adgre1*, *Mrc1*, and *Retnla*), neutrophils (*Cxcr2* and *Lcn2*), T cells (*Cd3e*, *Cd4*, *and Cd8a*), DCs (*Cd209a* and *Flt3*), and mast cells (*Cpa3*, *Mcpt4* and *Mrgprb1*). In addition, cluster 9 was a cluster of mixed proliferating cells (*Mki67*) (Figs. 1A and S2B–I). B cells and macrophages represented two major cell types in our scRNA-seq data. Four B cell clusters (clusters 0, 3, 8, 12) were detected, among which cluster 0 (named as “B cells_1”) displayed higher *H2-Oa* and *Cr2* expression (Fig. S3A and S3B), indicating the function of antigen presentation. *Spib* and *Ms4a1* were highly expressed in cluster 8 (Fig. S3C and S3D), suggesting that this population may be memory B cells. Cluster 12 had higher expression of *Ssr4* and *Xbp1*, which were the markers of plasma cells (Fig. S3E and S3F). Cluster 3 (B cells_2) had no obvious characteristics of classic B cell subtypes. We also detected three diverse subsets in macrophage populations and one subset of mixed macrophages and monocytes. Gene Ontology (GO) pathway enrichment showed that all these four clusters had increased expression of genes related to inflammatory responses, endocytosis, immune system process and chemotaxis, suggesting a possible role of these populations in inflammatory regulations (Fig. S3G–J).

**Figure 1:**
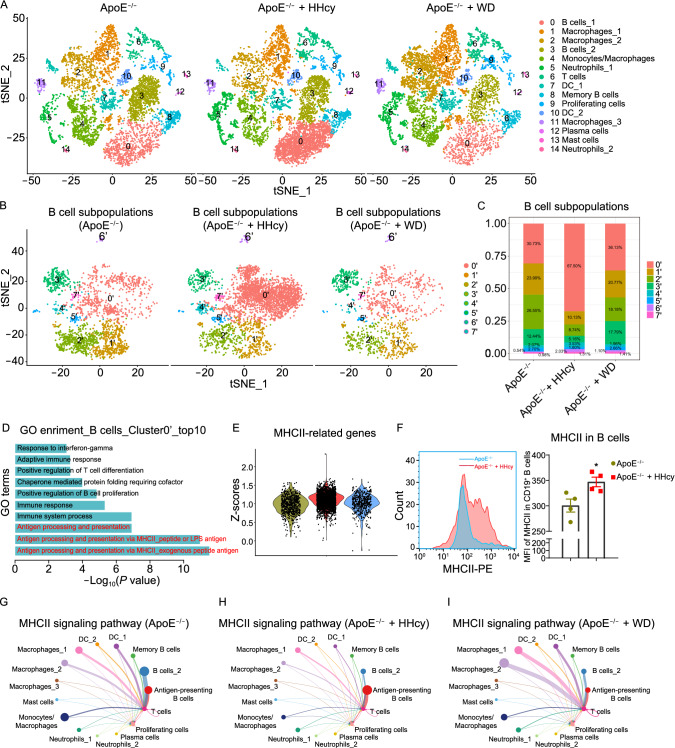
Antigen-presenting B cells play a dominant role in antigen presentation in HHcy-accelerated atherosclerosis. (A) tSNE plots showing color-coded cell clusters in the three groups. (B) tSNE plots displaying eight distinct B cell clusters. (C) Bar charts showing the percentages of B cell clusters in the three groups. (D) GO enrichment analyses of biological processes in cluster 0’. (E) Violin plots showing the scaled expression scores (Z-scores) of MHCII-related genes (*H2-Aa*, *H2-Eb1*, *H2-Ab1*, *H2-Ob*, *H2-Eb2*, *H2-Oa*, *H2-DMb2*, *Ctss*, *Cd74*, *H2-DMa*, and *Ctse*) across the three groups. (F) Flow cytometry analyses showing the levels of MHCII in CD19^+^ B cells from atherosclerotic aortas. (G–I) Circle plots showing MHCII signaling pathway in the three groups. Data represent mean ± SEM (*n* = 4 in (F)). * *P* < 0.05 by an unpaired 2-tailed *t* test (F).

Comparing the effects of HHcy with WD on ApoE^−/−^ mice, cell composition analyses of our scRNA-seq data showed that, B cells appeared to be the most abundant population (47.22%) in the vessels of HHcy ApoE^−/−^ mice, also showed the most significant increase compared with ApoE^−/−^ mice fed a CD, while macrophages/monocytes became the most abundant population in the WD ApoE^−/−^ group (Figs. 1A and S4A). Of note, in the HHcy ApoE^−/−^ group, a specific B cell cluster (cluster 0, B cells_1) was dramatically increased to 30.25% (ApoE^−/−^: 13.65%, WD ApoE^−/−^: 8.13%) (Figs. 1A and S4B). In addition, the proportion of neutrophils and T cells was increased, and the proportion of DCs and mast cells was decreased in the HHcy ApoE^−/−^ group (Fig. S4A and S4B). Focused analysis on proliferating cells (cluster 9) revealed that this cluster contains B cells, macrophages, monocytes, T cells and DCs (Fig. S4C and S4D). Among them, macrophages/monocytes became the majority of proliferating cells in the WD ApoE^−/−^ group, and CD4^+^/CD8^+^ T cells were highly increased in the HHcy ApoE^−/−^ group (Fig. S4E).

To confirm the findings from scRNA-seq, we further performed flow cytometry to detect the changes of immune cell (CD45^+^ gated) proportions in the atherosclerotic aortas (Fig. S5A). Consistent with the scRNA-seq data, significant increases in the proportions of B cells and CD3^+^ T cells, as well as decreases in the proportions of macrophages in the HHcy ApoE^−/−^ group were detected (Fig. S5B and S5C), compared with the ApoE^−/−^ group. We also observed similar decreases in the proportions of B cells, and the increases in the proportions of T cells and macrophages in the WD ApoE^−/−^ group (Fig. S5B–D). Collectively, our results present a comprehensive immune cell atlas in the aortas of HHcy ApoE^−/−^ mice, and reveal distinct immune cell compositions from those in the aortas of WD-fed ApoE^−/−^ mice.

To identify the possible function of B cells, which were one of the most abundant and dynamic cell populations in this mouse model, focused analysis of B cell populations was performed and identified eight B cell subsets (Fig. 1B). Among them, the proportion of cluster 0’ was dramatically increased from 30.73% to 67.50% in atherosclerotic aortas of the HHcy ApoE^−/−^ group compared with that of the ApoE^−/−^ group (Fig. 1C). Strikingly, GO pathway enrichment analyses showed that antigen presentation was the dominant pathways in cluster 0’ (top 3) (Fig. 1D). MHCII-related genes, such as *H2-Oa*, *H2-Eb2*, *H2-DMa* (genes encoding the components of the MHCII complex) and *Ciita* (class II transactivator, the master transactivator for MHCII molecule expression) were highly expressed in cluster 0’, compared with other B cell subpopulations (Fig. S6A–D). Average expression analyses by volcano plot revealed that cluster 0’ showed substantial differences from other B cells in terms of gene expression profile. These B cells highly expressed MHCII-related genes *H2-Aa*, *H2-Ab1*, *H2-Oa*, *H2-Ob*, *H2-Eb2*, *Cd74* and *Ciita* (Fig. S6E). High expression of chemokine receptors *Ccr6* and *Cxcr4* was also observed in cluster 0’ (Fig. S6E). These results indicate that MHCII-mediated antigen presentation might be one of the most important function in cluster 0’, and migration from peripheral sites might be possible source of these B cells. B cells_1 (cluster 0 in Fig. 1A) was the majority of cluster 0’ in focused analysis. Therefore, we annotated these B cells (cluster 0 in Fig. 1A or cluster 0’ in Fig. 1B) as “antigen-presenting B cells”.

Interestingly, inside this antigen-presenting B cell cluster, the expression of MHCII-related genes was also highly upregulated in the HHcy ApoE^−/−^ group specifically (Figs. 1E and S6F–I). Flow cytometry further confirmed this finding in the protein level (Fig. 1F). In addition, activated CD4^+^ T cells (CD4^+^ IFN-γ^+^) were also highly increased in the aortas from HHcy ApoE^−/−^ mice (Fig. S6J). These results were consistent with the results from the scRNA-seq that the proportions of T cells and proliferating T cells were highly increased. Moreover, we performed focused analysis on cluster 6 in Fig. 1A and revealed that CD4^+^ T cells were the most dynamic cluster with increases in the HHcy ApoE^−/−^ group (Fig. S6K–M). Additionally, type 2 innate lymphoid cells (ILC2), natural killer (NK) cells and γδT cells with small numbers were also identified in this cluster (Fig. S6K–M). Taken together, our results demonstrate increases in cell numbers and MHCII-related gene expression of antigen-presenting B cells in the HHcy ApoE^−/−^ group. The upregulation of MHCII signaling in B cells may further increase CD4^+^ T cell activation in HHcy-AA.

MHCII-mediated antigen presentation is a typical form of cell-cell contact between professional antigen presenting cells and CD4^+^ T cells. To explore the potential intercellular communications among the identified immune cells and evaluate the role of antigen-presenting B cells, we performed cell-cell contact analysis using major immune cell groups via CellChat (Jin, et al., 2021). We identified both five outgoing and incoming communication patterns, and revealed B cells as a major source of MHCII signaling (outgoing pattern 2), while T cells served as a prominent receiver of this signaling (incoming pattern 2) in the HHcy ApoE^−/−^ group (Fig. S7A–C). To further understand the role of MHCII signaling in HHcy-AA, we analyzed MHCII pathway networks among all finer immune cell clusters. While multiple macrophages, DCs and B cell subsets displayed intermediate and strong communication with T cells in the control ApoE^−/−^ group and the WD ApoE^−/−^ group, we observed a dominant interaction between antigen-presenting B cells and T cells in the HHcy ApoE^−/−^ group (Fig. 1G–I). These results suggest that antigen-presenting B cells are the most dominant source of MHCII signaling in HHcy-AA.

MHCII-mediated antigen presentation between B and T cells requires the help of costimulatory molecules. We noticed that the inducible T-cell costimulator (ICOS) pathways was also enriched in the HHcy ApoE^−/−^ group (Fig. S7A–C). Similarly, antigen-presenting B cells were revealed as an important source of the ICOS pathways in the HHcy ApoE^−/−^ group, but not in the other two groups (Fig. S7D–F). We also observed increases in the expression of MHCII-related genes (*H2-Oa)* in antigen presenting B cells and the T cell receptor (TCR)-related gene *Cd4* in T cells were highly upregulated (Fig. S7G and S7H). Costimulatory molecules (*Icosl* in antigen-presenting B cells, *Icos* in T cells) were also elevated in the HHcy ApoE^−/−^ group (Fig. S7I and S7J). Flow cytometry showed the similar changes of MHCII and ICOSL in aortic B cells (Fig. S7K and S7L). In addition, plasma IFN-γ and IL-2, which are mainly secreted from activated T cells, were significantly increased in HHcy ApoE^−/−^ mice (Fig. S7M and S7N). To further explore the localization of the increased B cells and CD4^+^ T cells, we performed confocal imaging analysis. The results showed that B cells and CD4^+^ T cells mainly localized at the adventitia of the vessels, and artery tertiary lymphoid organs (ATLOs) (CD19^+^CD4^+^GL7^+^) were observed in the aortas from HHcy ApoE^−/−^ mice (Fig. S7O), but not in those from ApoE^−/−^ mice and WD ApoE^−/−^ mice (data not shown). Therefore, these results indicate that MHCII and costimulatory molecule signaling might be critical for the interaction between B cells and T cells in the adventitia of HHcy-AA.

To elucidate the underlying mechanisms, splenic CD19^+^ B cells were isolated from C57BL/6J mice and cultured with or without Hcy (100 μmol/L) for 24 h, and then these cultured B cells were subjected to transcriptome sequencing analysis. The results showed that the expression of MHCII-related genes, including genes encoding MHCII components and the upstream master regulator of MHCII expression, *Ciita*, was significantly upregulated by Hcy (Fig. 2A). Quantitative PCR validated these data (Fig. S8A). Flow cytometry and Western blot showed that Hcy significantly elevated the expression of MHCII and CIITA (Figs. 2B and S8B). The results from confocal images also showed an elevation in MHCII translocation to the plasma membrane of B cells (Fig. 2C). These data demonstrate that Hcy upregulates MHCII expression in B cells at both the gene and protein levels *in vitro*. To test the function of Hcy-increased MHCII in B cells, we designed a B cell-T cell coculture system to measure the ability of MHCII-involved antigen processing and presentation *in vitro* (Fig. S8C), which is widely used (Deng, et al., 2013). B cells pretreated with Hcy significantly increased the secretion of IL-2 and IFN-γ from CD4^+^ T cells (whose TCR has antigen specificity for chicken ovalbumin, OVA) under stimulation with OVA (Fig. S8D and S8E). In the coculture system, we also detected an increase in the costimulatory molecules *Cd40*, *Cd80*, and *Cd86* in B cells (Fig. S8F), and *Cd40lg*, *Cd2*, and *Cd27* in CD4^+^ T cells (Fig. S8G). However, Hcy had little effect on T cell activation mediated by DCs and macrophages in their coculture system (Fig. S8H–K), suggesting a specific effect of Hcy on B cells as functional antigen-presenting cells.

**Figure 2:**
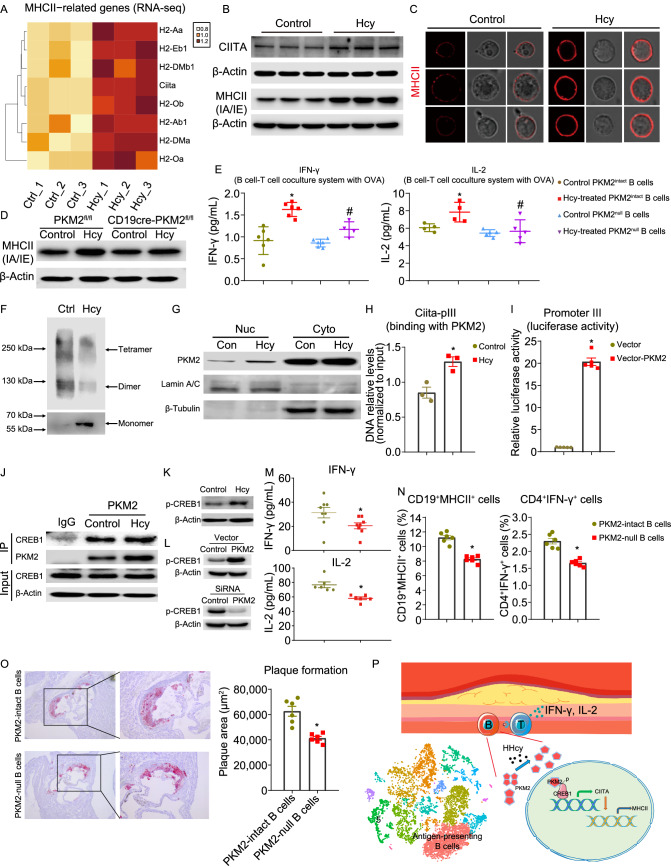
PKM2-induced antigen presentation is critical for B cell-mediated T cell activation in HHcy mice. (A–C) Purified CD19^+^ splenic B cells from C57BL/6J mice were cultured with or without Hcy (100 μmol/L) for 24 h. (A) Heatmap showing the mRNA levels of MHCII-related genes identified by RNA-seq. (B) The CIITA and MHCII expression and quantification were analyzed via Western blot. (C) Representative images showed staining of MHCII on B cells. (D) Purified CD19^+^ splenic B cells from PKM2^fl/fl^ and CD19CrePKM2^fl/fl^ mice were cultured with or without Hcy (100 μmol/L) for 24 h. MHCII protein expression was analyzed and quantified via Western blot. (E) Splenic B cells purified from PKM2^fl/fl^ and CD19CrePKM2^fl/fl^ mice (showed as PKM2^intact^ and PKM2^null^ B cells, respectively) were pretreated with or without 100 μmol/L Hcy for 24 h and then cocultured with OVA-specific CD4^+^ T cells. OVA was added for another 48 h. IFN-γ and IL-2 secretion was analyzed via ELISA. (F–H) Purified CD19^+^ splenic B cells from C57BL/6J mice were cultured with or without Hcy (100 μmol/L) for 24 h. (F) B cell lysates were cross-linked with glutaraldehyde and subjected to Western blot for detecting PKM2 oligomer states. (G) Nucleic and cytoplasmic proteins from B cells were extracted and subjected to Western blot for PKM2 detection. Lamin A/C is a marker of nucleic proteins. β-Tubulin is a marker of cytoplasmic proteins. (H) The DNA levels of PKM2-binding Ciita promoter III were measured via ChIP using an anti-PKM2 antibody and qPCR. (I) The transcriptional activity of *Ciita* promoter III with or without PKM2 overexpression was detected via the dual-luciferase system. (J) Purified CD19^+^ splenic B cells from C57BL/6J mice were cultured with or without Hcy (100 μmol/L) for 24 h, and then cell extracts were subjected to coimmunoprecipitation with an antibody against PKM2. IgG servers as a negative control. Binding of PKM2 and CREB1 was detected and quantified via Western blot. (K) Purified CD19^+^ splenic B cells from C57BL/6J mice were cultured with or without Hcy (100 μmol/L) for 24 h. Phosphorylation of CREB1 in Hcy-treated B cells was detected via Western blot with an antibody against p-CREB1. (L) Phosphorylation of CREB1 in primary B cells with *Pkm2* overexpression by plasmid transfection (1.25 μg/mL, 48 h) or *Pkm2* knockdown by siRNA (50 pmol/mL, 48 h) was detected via Western blot. (M–O) Eight-week-old female ApoE^−/−^ mice were injected with antibodies against CD19 and CD20 to deplete B cells. After 7 days, splenic B cells from PKM2^fl/fl^ or CD19crePKM2^fl/fl^ mice were transferred into B cell-deficient ApoE^−/−^ mice, and drinking water supplemented with Hcy (1.8 g/L) was provided for 28 days. (M) Plasma IFN-γ and IL-2 were analyzed via ELISA. (N) Flow cytometry analyses showing the percentages of CD19^+^MHCII^+^ cells and CD4^+^IFN-γ^+^ cells in atherosclerotic aortas. (O) Representative images showing cross-sections of aortic roots stained with Oil Red O, to assess and quantify plaque formation and lipid deposition. (P) Graphic model of this work. Antigen-presenting B cells appear to be the most abundant populations in immune cell atlas of HHcy-AA. Hcy increases the accumulation of PKM2 monomer and its nuclear translocation. Nuclear PKM2 interacts with and phosphorylates CREB1, induces CIITA-MHCII expression, and further increases B cell-T cell interaction, therefore activates CD4^+^ T cells. Data represent the mean ± SEM (*n* = 3 in (H), *n* = 4–6 in (E), *n* = 6 in (N and O), *n* = 6–8 in (M)). *P < 0.05 by an unpaired 2-tailed *t* test (H, I, M–O). * or # *P* < 0.05 by one-way ANOVA followed by Tukey’s test for multiple comparisons (E). *Indicates the comparison with the Control group (H), the vector group (I) , or the PKM2^intact^ B cell group (M–O), #Indicates the comparison with the Hcy-treated PKM2intact B cells group (E).

We next sought to explore possible mechanisms involved in the regulation of MHCII expression in B cells by Hcy. PKM2 is a key metabolic enzyme in glycolysis and also act as a protein kinase to regulate gene expression while translocating into the nucleus. We previously reported that Hcy increases the expression of PKM2 in B cells (Deng, et al., 2017). To elucidate whether PKM2 is involved in Hcy-induced MHCII expression in B cells, we employed B cell-specific PKM2-deficient (CD19cre-PKM2^fl/fl^) mice and isolated splenic B cells. Protein expression of PKM2 was efficiently depleted in these B cells (Fig. S9A), whereas *Cd19* in B cells and PKM2 in T cells and macrophages were not affected (Fig. S9B–D). As expected, B cell-specific PKM2 deficiency significantly reduced the Hcy-induced upregulation of MHCII expression (Figs. 2D and S9E). Confocal imaging analysis confirmed the decreases in MHCII on the plasma membrane (Fig. S9F). Consistently, PKM2 deficiency also reversed the upregulation of the mRNA levels of MHCII-related genes in the Hcy group (Fig. S9G). We next tested whether PKM2 depletion affects the function of antigen presentation. While control B cells pretreated with Hcy promoted IFN-γ and IL-2 production by OT-II CD4^+^ T cells, PKM2 deficiency in B cells significantly restricted the activation of OT-II CD4^+^ T cells (Fig. 2E). Furthermore, the upregulation of B cell costimulatory molecules (*Cd40*, *Cd86*) was also inhibited by PKM2 deficiency (Fig. S9H). These results demonstrate that PKM2 is essential for Hcy-upregulated MHCII expression in B cells, and further B cell-mediated CD4^+^ T cell activation.

In addition, previous studies have established that dimeric and monomeric forms of PKM2 can translocate into nucleus and regulate gene expression directly. ScRNA-seq data showed no changes in glycolysis, the tricarboxylic acid (TCA) cycle and the pentose phosphate pathway which are regulated by PKM2, in aortic B cells from atherosclerotic mice (Fig. S9I). Both glycolysis inhibitor 2-DG and PKM2 enzymatic inhibitor shikonin (SKN) had no effect on Hcy-induced MHCII expression (Fig. S9J). Therefore, we speculated that changes in the oligomeric state and the non-enzymatic nuclear function of PKM2 might regulate MHCII-related gene expression. To confirm this hypothesis, we detected the oligomerization form of PKM2 in B cells. The results showed that Hcy promoted the transformation of PKM2 from the tetramer and dimer to the monomer (Fig. 2F), and significantly increased the translocation of PKM2 proteins into the nucleus (Fig. 2G). Moreover, PKM2 activator TEPP-46 (10 μmol/L), which stabilizes the PKM2 tetramer and prevents the accumulation of its dimeric or monomeric forms and nuclear translocation, significantly downregulated the expression of MHCII in B cells (Fig. S9K). Collectively, these results indicate that Hcy promotes the accumulation of monomeric forms of PKM2 and subsequently PKM2 nuclear translocation may possibly participate in the regulation of MHCII-related gene expression, via a non-metabolic role in B cells.

To elucidate how PKM2 regulates the gene expression of MHCII, we focused on CIITA that was highly upregulated by Hcy both *in vivo* and *in vitro*. CIITA is a master transactivator of MHCII expression and is definitely required for the transcription of MHCII-related genes. Previous studies have found three promoters located within an upstream region of the *Ciita* gene that control the transcription of *Ciita* in mice (Kobayashi, et al., 2012) (Fig. S10A). We detected the presence of transcripts induced by promoters III and IV (*Ciita*-pIII and *Ciita*-pIV) but not transcripts induced by promoter I in B cells (*Ciita*-pI) (Fig. S10B). Of note, Hcy specifically increased the expression of *Ciita*-pIII-induced transcripts (Fig. S10B). We next performed chromatin immunoprecipitation (chIP) assays and revealed the binding of PKM2 to *Ciita*-pIII, while Hcy enhanced this binding process (Fig. 2H). A dual-luciferase (firefly and Renilla) reporter assay system revealed that PKM2 overexpression into HEK-293T cells significantly promoted transcription induced by *Ciita*-pIII but not *Ciita*-pIV (Figs. 2I and S10C). In primary B cells, transfection of the PKM2 plasmid also significantly increased the CIITA at the mRNA and protein levels, as well as its downstream MHCII-related genes (Fig. S10D and S10E). Collectively, these results demonstrate that Hcy-increased PKM2 induces CIITA expression via binding to *Ciita*-pIII.

It remains unclear whether PKM2 binds to *Ciita*-pIII directly or indirectly. Although previous reports did not discover any DNA binding domain or motif for PKM2, several transcription factors linking PKM2 and targeted genes have been suggested. We analyzed the relative scores of potential transcription factors targeting *Ciita* using ChIP-Atlas (Oki, et al., 2018), and revealed interferon regulatory factor 4 (IRF4) and cyclic AMP-responsive element-binding protein 1 (CREB1) as the top 2 candidates (Fig. S10F), although neither of these proteins have been reported to interact with PKM2. We next tested whether IRF4 and CREB1 were involved in these processes. The results from co-IP assays showed that PKM2 directly bound to CREB1, which was significantly promoted by Hcy (Fig. 2J). However, the binding of IRF4 and PKM2 was not detected (data not shown). CREB1 is an important regulator of *Ciita* and can directly bind to *Ciita*-pIII (van der Stoep, et al., 2002), and the activity of CREB1 as a transcription factor depends on its phosphorylation state (Altarejos, et al., 2011). Of note, the upregulation of CREB1 phosphorylation in B cells by Hcy was observed (Fig. 2K), consistent with a previous report that Hcy induces CREB phosphorylation in hepatocytes (HepG2) (Woo, et al., 2006). More importantly, in the scRNA-seq data, several target genes of CREB1 (*Ap2b1*, *Eif2b1*, *Noc4l*, and *Haus5*) were highly upregulated in antigen-presenting B cells in the HHcy ApoE^−/−^ group, however, they were not affected by WD (data not shown). PKM2 knockdown and overexpression also dramatically downregulated and upregulated the phosphorylation of CREB1, respectively (Fig. 2L). Taken together, these results reveal a novel pathway that Hcy-increased nuclear PKM2 directly binds to CREB1, phosphorylates CREB1, and further PKM2-CREB1 complex binds to CIITA promoters and induces CIITA and its downstream MHCII expression in B cells.

To further elucidate the role of PKM2 in regulating antigen presentation *in vivo*, we first used an OT-II mouse model to evaluate the antigen presentation process between B and T cells (Fig. S10G). We applied anti-CD19 and anti-CD20 monoclonal antibodies to deplete B cells in OT-II mice for 7 days. B cells were efficiently depleted in the peripheral blood (Fig. S10H), without affecting the other professional antigen presenting cells (DCs and F4/80^+^ cells) (Fig. S10I). PKM2-intact or PKM2-null B cells were then transferred into these recipient mice. At the same time, OVA was injected, and Hcy-containing drinking water was provided for another 14 days. Results showed the expression of MHCII-related genes was lower in PKM2-null B cells (Fig. S10J). ELISA and flow cytometry showed that mice PKM2-null B cell transferring lowered T cell activation (Figs. 2M, S10K and S10L). The costimulatory molecules *Cd40*, *Cd80*, and *Cd86* in splenic B cells and *Cd40lg*, *Cd27*, and *Cd2* in splenic CD4^+^ T cells from recipient mice with PKM2-null B cells were also downregulated (Fig. S10M and S10N). These data confirmed an essential role of PKM2 of B cells in regulating CD4^+^ T cell activation *in vivo*.

To further confirm the role of B cell PKM2 in HHcy-accelerated atherosclerosis, we applied the same adoptive transferring strategy in the early stage atherosclerosis mouse model, in which ApoE^−/−^ mice were supplemented with or without Hcy-containing water for 28 days. Flow cytometry results showed that PKM2-null B cell transferring lowered the numbers of B cells and T cells in HHcy-AA, but the numbers of macrophages were slightly increased (Fig. S11A–D). Further analysis revealed that the ability of B cell antigen presentation (CD19^+^MHCII^+^) and activation of CD4^+^ T cells (CD4^+^IFN-γ^+^) was significantly decreased in the PKM2-null B cell transferring group (Fig. 2N). More importantly, Oil Red O staining showed significant decrease in plaque formation by PKM2-null B cell transferring (Fig. 2O). Summarily, these data demonstrate that nuclear PKM2 is critical for B cells to activate CD4^+^ T cells, and mediates HHcy-accelerated atherosclerosis, at least in part by inducing MHCII expression in B cells (Fig. 2P).

While we provided a comprehensive immune cell atlas in HHcy-AA and highlighted the role of antigen presentation in local B cells, the limitations of our study should be acknowledged. First, the proportions of B cells are different between our study and a previous study (Winkels, et al., 2018), which might be due to the background, ages of the mouse, the time of specific-diet feeding, and the maintaining environment. The estimation of immune cells in different models following the same procedure is necessary. Second, potential antigens presented by antigen-presenting B cells in HHcy-AA are not identified yet.

In summary, we first provide a comprehensive immune cell landscape in HHcy-AA and reveal that aortic B cells mainly function as antigen-presenting cells with dominance in atherosclerosis, especially those accelerated by HHcy. Moreover, the role of nuclear PKM2-CREB1-CIITA axis in Hcy-induced MHCII expression in B cells was firstly clarified. This finding might provide new insights into HHcy-accelerated atherosclerosis and novel targets for the prevention and treatment of this disease.

## Supplementary Information

Below is the link to the electronic supplementary material.

Supplementary file1 (PDF 76223 KB)
