# Supplementary material for: Single-cell RNA sequencing reveals B cell-T cell interactions in vascular adventitia of hyperhomocysteinemia-accelerated atherosclerosis
Source: Protein Cell. 2022 Feb 17;13(7):540–7. doi: 10.1007/s13238-021-00904-0 (PMC9226200; doi:10.1007/s13238-021-00904-0)
Supplement: Supplementary file 1 — Supplementary file1 (PDF 76223 KB) [file 13238_2021_904_MOESM1_ESM.pdf]

**ScRNA-seq reveals B cell-T cell interactions in HHcy-accelerated atherosclerosis**

Xiaolong Ma, Jiacheng Deng, Lulu Han, Yuwei Song, Yutong Miao, Xing Du, Guohui Dang, Dongmin Yang, Bitao Zhong, Changtao Jiang, Wei Kong, Qingbo Xu, Juan Feng*, Xian Wang*

# SUPPLEMENTAL MATERIAL

## Detailed Methods

### *Mice and animal experiments*

C57BL/6J mice were purchased from the Animal Center of Peking University Health Science Center (Beijing, China) and maintained under specific pathogen-free (SPF) conditions. For HHcy animal model, 8-week-old female $ApoE^{-/-}$ mice were fed a normal chow diet and drinking water supplemented with or without 1.8 g/L Hcy (Sigma Aldrich) for 4 weeks. For WD $ApoE^{-/-}$ model, 8-week-old female $ApoE^{-/-}$ mice were fed a WD for 4 weeks. $PKM2^{fl/fl}$ mice (Jax-24048, on a B6129SF1/J background) and CD19-cre transgenic mice (Jax-006785) were purchased from The Jackson Laboratory (Bar Harbor, ME, USA). CD19cre-$PKM2^{fl/fl}$ mice were generated by crossing $PKM2^{fl/fl}$ mice and CD19-cre transgenic mice with help from Shanghai Biomodel Organism and Technology Development (Shanghai, China) in our laboratory. CD19cre-$PKM2^{fl/fl}$ mice were maintained under SPF conditions.

All animal procedures were reviewed and approved by the Institutional Animal Care and Use Committee of Peking University Health Science Center in accordance with the U.S. Department of Agriculture, International Association for the Assessment and Accreditation of Laboratory Animal Care,

and National Institutes of Health (NIH; Bethesda, MD, USA) guidelines.

### *Isolation of single $CD45^+$ live cells from the aortas*

Aortas were isolated and digested as previously described (Cai, et al., 2020; Winkels, et al., 2018). Briefly, after sacrificed mice, we firstly perfuse the aortas by PBS, and then harvested whole aortas by pulling of all of the adipose tissue and collecting all aortic layer including the adventitia into RPMI 1640 medium containing 10% (v/v) fetal bovine serum (FBS) on ice, we cut the aortas into fine pieces and digested in HBSS containing 450U/mL Collagenase I (Sigma Aldrich), 250 U/mL Collagenase XI (Sigma Aldrich), 120 U/mL Hyaluronidase (Sigma Aldrich) and 120 U/mL DNase I (Warthington) at 37℃ with reciprocal shaking (60 rpm/min) for 1 hour. Detached cells were then filtered through a 40 μm cell strainer (BD Biosciences), and red cells were excluded by Red Cell Lysis Buffer. Subsequently the remaining cells were washed with 37℃ pre-warmed 1640 containing 10% FBS (500g, 5min) and suspended with PBS. Cells were then stained with FITC anti-mouse CD45 antibody (Biolegend, 103108) (1:100), Hoechst 33342 (Invitrogen, H3570) (1:1000) for 10 min at room temperature, then stained with LIVE/DEAD$^{TM}$ Fixable Near-IR Dead Cell Stain Kit (Invitrogen, L34975) for 5 min at room temperature. After PBS washing, cells were suspended in PBS. Single $CD45^+$ live cells

(Hoechst$^{+}$/Dead Cell Stain$^{-}$/CD45$^{+}$) were sorted into PBS with 0.04% BSA using a BD FACS SymphonyS6 (BD Biosciences) before single cell RNA-sequencing. Antibodies used were shown in Online Table I.

***Single-cell RNA-sequencing and data processing***

We performed single-cell RNA-sequencing of samples from the ApoE$^{-/-}$ group, the HHcy ApoE$^{-/-}$, and the WD ApoE$^{-/-}$ group separately. A Chromium Single Cell 3' Reagent Kits v3 was used and Standard 10X Chromium Single Cell 3' v3 (10X Genomics GemCode Technology) protocols were followed for scRNA-seq. Briefly, single cells with specific 10X Barcodes and unique molecular identifier (UMI) were generated by partitioning the cells into Gel Bead-In-Emulsions. cDNA library was generated and sequenced with Novaseq (S4) 6000 (PE150). Raw sequencing data were processed using Cell Ranger (version 2.2.0) to demultiplex raw data and generate FASTQ files, aligned, and counted with Cell Ranger pipelines. ScRNA-seq detected 7512 cells with a mean of 96347 reads and a median of 2152 genes per cell in the ApoE$^{-/-}$ group, 9753 cells with a mean of 79160 reads and a median of 2134 genes per cell in the HHcy ApoE$^{-/-}$ group, and 8557 cells with a mean of 81780 reads and a median of 2371 genes per cell in the WD ApoE$^{-/-}$ group (Online Fig I C).

For further analyses and visualization, R package Seurat (version 3.2.2) (Stuart, et al., 2019) was used. Seurat objects of two samples were created and then integrated as one for following processing and analyses. Briefly, gene features expressed in at least 3 cells, and cells with at least 200 and less than 7000 detected genes, less than 10% mitochondrial counts were kept. Then, Gene expression were Log-normalized. 2000 most highly variable genes were selected and all genes were scaled. 6030 cells in the ApoE$^{-/-}$ group, 8101 cells in the HHcy ApoE$^{-/-}$ group, and 6683 cells in the WD ApoE$^{-/-}$ group were remained. Principle component analysis was then performed and the first 30 principle components with a resolution of 0.2 were used for unbiased clustering. Clusters were visualized with t-distributed stochastic nearest neighbor embedding (tSNE). The markers for every cluster compared to all remaining cells were identified by "FindAllMarkers" (min.pct=0.25, logfc.threshold=0.25, only.pos=TRUE), and were used as differently expressed genes (DEGs) in following analyses.

After cell type identified, we found that cluster 10 and 12 were fibroblasts (highly expressing fibroblast markers *Dcn* and *Mgp*) and nonimmune cells (no *Ptprc* expression), respectively. Therefore, cluster 10 and 12 were excluded and remained cells were pre-processed and unbiased clustered again in a similar way as described above. Finally, a total of 20309 cells (ApoE$^{-/-}$: 5979; HHcy ApoE$^{-/-}$: 7925; WD ApoE$^{-/-}$: 6405) were remained, and 15 clusters were identified.

***Focused analysis on specific cell clusters***

For focused analysis on B cells, cluster 0, 3, 8, 12 in Fig 1C were extracted by "subset" and unbiased clustered again with a resolution of 0.4.

For focused analysis on T cells, cluster 6 in Fig 1C was extracted by "subset", then possible B cells, monocytes and macrophages were excluded by using 'subset=Cd79a>0|Cd14>0|Mafb>0,

invert=TRUE'. Then the remaining cells were unbiased clustered again with a resolution of 0.4. For focused analysis on proliferating cells, cluster 9 in Fig 1C was extracted by "subset" and unbiased clustered again with a resolution of 0.2.

### *Cell-cell contact analysis*

For cell-cell contact analysis, R package CellChat (3.0.2) (https://github.com/sqjin/CellChat) (Jin, et al., 2021) was used. Briefly, two sample objects were extracted from merged Seurat object by "subset". After nominating clusters as cell types, CellChat object was created by "createCellChat". For further cell-cell contact analysis, CellChat database "CellChatDB.use" was created by "subsetDB" (search= "Cell-Cell Contact"). Then, overexpressed genes were identified by "identifyOverExpressedGenes", overexpressed interactions were identified by "identifyOverExpressedInteractions" and mouse dataset were selected by "projectData(cellchat, PPI.mouse)". Finally, communication probability of MHCII pathway was computed by "computeCommunProb" and probable pathways were computed by "computeCommunProbPathway". For visualization, alluvial plots were created by "netAnalysis_river", circle plots was created by "netVisual_aggregate", and heatmap was created by "netVisual_signalingRole".

### *Pathway enrichment analysis with Gene Ontology (GO)*

For functional pathway analyses, DAVID (Huang da, et al., 2009) was used to perform enrichment analyses of gene ontology biological process (GOBP). DEGs of selected cell clusters were used as input data. Top10 enrichment pathways were visualized by R (4.0.3).

### *Splenic B cell isolation and culture*

Mouse splenic B cells were isolated and purified by positive magnetic cell sorting system using CD19 mouse microbeads (Miltenyi Biotec, 130-121-301) with standard procedure. Purified $CD19^+$ B cells were cultured in RPMI 1640 medium (Thermo Fisher Scientific, c11875500BT) supplemented with 10% FBS (Gemini Bio-Products, 900-108) and maintained with LPS (0.1 µg/ml, as basic stimulation for B cells; Millipore-Sigma, L2880). B cells were treated with or without 100 µmol/L Hcy for 24 hours.

### *Quantitative Real-time PCR (qPCR)*

Total RNA of purified or cultured cells were extracted by the TRIzol reagent method (Thermo Fisher Scientific, 15596018), then reverse-transcribed to cDNA and amplified using the reverse transcription (RT) system: 5X All-In-One RT MasterMix Kit (Applied Biological Materials, G490). qPCR was then used to measure the cDNA content with an Mx3000 Multiplex Quantitative PCR system (Stratagene) and SYBR Green Influorescence. Expression of the β-actin served as an internal control. The primer sequences were shown in Online Table II.

### *Bulk RNA sequencing*

Splenic $CD19^+$ B cells were cultured with or without Hcy (100 μM) for 24 hours. Total RNA from cultured B cells was extracted as described above. Detailed sample preparation and RNA sequencing as described previously (Miao, et al., 2020).

### *Western Blot*

Proteins were extracted from cells by RIPA lysis buffer, and the lysate were resolved with 10% SDS-polyacrylamide gel electrophoresis (PAGE) and transferred to nitrocellulose membranes, which were then blocked with 5% (w/v) fat-free milk for 1 hour and incubated with antibodies at 4℃ overnight, followed by an incubation of peroxidase-conjugated anti-rabbit, mouse, or goat IgG according to the species sources of primary antibodies. Antibodies were shown in Online Table I.

### *ELISA*

Titers of IL-2 and IFN-γ in plasma from mice and B-T cell co-culture system were determined by ELISA using mouse-specific IL-2 (NEOBIOSCIENCE, EMC002) and IFN-γ (NEOBIOSCIENCE, EMC101g) ELISA kit according to manufacturer’s protocol.

### *Flow cytometry*

Briefly, purified or cultured cells were washed with PBS twice, and incubated with indicated antibodies for 1 hour. Then, cells were washed twice with PBS and analyzed with an FACS Calibur Flow Cytometer (BD Bioscience). Antibodies were shown in Online Table I.

### *Immunofluorescence and Oil Red O staining*

For immunofluorescence staining of specific immune cells in mouse aortas and Oil Red O staining for lesion area visualization, the aortas were first collected, washed with PBS and fixed in 4% paraformaldehyde (PFA) (Santa Cruz, sc-281692) at 4℃ for 2-3 hours, and then dehydrated in 30% sucrose solution (BDH AnalaR, 102747E) overnight. The aortas were then embedded in OCT and cut into 10-μm sections using a CryoStar Cryostat (Thermo Scientific). After blocking with 5% BSA, the sections were stained with primary antibodies overnight at 4℃. Hoechst (Invitrogen, H3570) or DAPI (Molecular Probe, D1306) was added and stained for 5 minutes.

For immunofluorescence staining of MHCII in B cells, cultured B cells were collected and washed with PBS twice. Anti-MHCII antibody (conjugated to PE) was added and incubated for 30 minutes at room temperature. Stained B cells were then washed with PBS twice and transferred onto Poly-D-Lysine-coated plates. The list of antibodies used was provided in Online Table I.

### *In Vitro Antigen Presentation Assay*

For B-T cell co-culture system (Deng, et al., 2013), splenic $CD19^+$ B cells isolated from C57BL/6J mice, $PKM2^{fl/fl}$ mice and $CD19\text{-}crePKM2^{fl/fl}$ mice were cultured in 12-well plates ($1x10^6$ cells/well) with or without 100 μM Hcy for 24 hours as indicated. $CD4^+$ T cells isolated from OT-II mice using

CD4 microbeads (Miltenyi Biotec) were then added to the plates ($2x10^6$ cells/well). 500 μg/ml OVA was added into co-culture system for 48 hours, followed by cells and supertanant harvesting.

For DC-T cell co-culture system and macrophage-T cell co-culture system, DCs were sorted from peripheral blood mononuclear cells (PBMCs) of C57BL/6J mice by FACS ($CD11c^+$), and peritoneal macrophages were collected by PBS (5% BSA). The co-culture systems were the same as described above.

***PKM2 Oligomerization Assay***

Splenic $CD19^+$ B cells were isolated from C57BL/6J mice and cultured with or without Hcy (100 μM) for 24 hours. The whole cell lysates (4 mg/ml) were then cross-linked with glutaraldehyde (0.025%) for 3min at 37℃ terminated with Tris-HCl (50 mM, pH 8.0). 5X loading buffer without β-mercaptoethanol were then added to the cross-linked proteins and incubated at 100℃ for 5 minutes. Subsequently, western blot was performed.

***Nuclear and cytoplasmic extraction***

Splenic $CD19^+$ B cells were isolated from C57BL/6J mice and cultured with or without Hcy (100 μM) for 24 hours. Nucleic and cytoplasmic proteins in B cells were then extracted by NE-PER™ Nuclear and Cytoplasmic Extraction Reagents (Thermo Fisher Scientific, 78833) according to manufacturer's protocol.

***Chromatin immunoprecipitation (ChIP)***

Splenic $CD19^+$ B cells were isolated from C57BL/6J mice and cultured with or without Hcy (100 μM) for 24 hours. Pierce™ Magnetic ChIP Kit (Thermo Fisher Scientific, 26157) and PKM2 antibody (Cell Signaling Technology, 4053S) was used to perform ChIP. The negative control was DNA immunoprecipitated by normal rabbit IgG, and the positive control was Gapdh immunoprecipitated by anti-RNA polymerase II antibody.

***ChIP-Atlas***

Based on published reports, we collected 15 potential transcriptional factors targeting Ciita (STAT3, CREB1, HIF1A, CEBPB, IRF4, ATF1, CTNNB1, BLMP1, NFKB1, NFKB2, GATA1, GATA2, STAT4, TBX2, and TBX5) as input for "Choose Antigen". Distance from TSS: ± 1 kb. Relative scores were analyzed via ChIP-Atlas (http://chip-atlas.org/target_genes) (Oki, et al., 2018).

***Co-immunoprecipitation (co-IP)***

Splenic $CD19^+$ B cells were isolated from C57BL/6J mice and cultured with or without Hcy (100 μM) for 24 hours. B cells were then lysed in cold RIPA buffer, incubated with PKM2 antibody (Cell Signaling Technology, 4053S), and immunoprecipitated with protein A/G agarose beads (Santa Cruz

Biotechnology). Rabbit IgG was used as a negative control. The precipitated proteins were resolved by 10% SDS-PAGE and then incubated with indicated antibodies.

***Plasmid construction and transfection***

Mouse *Pkm2* was amplified from B-cell cDNA with primers containing 5’ HindIII and 3’ EcoRI restriction sites. 3×Flag-pCMV vectors were cut with HindIII and EcoRI, and then ligated with the PCR product using Ligation-Free Cloning System (abm, E001). The constructed Pkm2 plasmid were transformed into cells and amplified. For the transfection, lipo8000 transfection agent (Beyotime Biotechnology) was used. Detailed sequences were shown in Online Table II.

***The Dual-Luciferase system***

Firefly luciferase reporter vectors with Ciita promoter III or Ciita promoter IV were designed and constructed by GENEWIZ (Suzhou, China). A Renilla luciferase control vector (Promega) were used as internal control. HEK-293T cells were used for transfection. After transfection for 8 hours using Lipo8000™ (Beyotime Biotechnology) transfection reagent, vector or vector with Pkm2 were transfected into cells for another 48 hours. Luciferase assays were performed using the dual-luciferase assay system (Promega). Firefly and Renilla luciferase activities were measured by Veritas microplate illuminometer (Turner Biosystems)

***Small interfering RNA***

Small interfering RNA (siRNA) targeting Pkm2 (5’-CTTGCAGCTATTCGAGGAA-3’) were designed by and purchased from RiboBio (Guangzhou, China). For the transfection, Lipo8000™ (Beyotime Biotechnology) transfection reagent was used.

***Adoptive transferring***

For adoptive B-cell transfer experiments, 8-week-old OT-II mice were injected with antibodies against CD19 (75 μg/mouse) and CD20 (50 μg/mouse) to deplete B cells. After 7 days, splenic $CD19^+$ B cells ($2X10^7$ cells/mouse, iv) from $PKM2^{fl/fl}$ or $CD19crePKM2^{fl/fl}$ mice were transferred into B cell-deficient OTII-mice, and NP-OVA (100 μg, ip) was injected every 7 days and drinking water with Hcy (1.8 g/L) were supplemented for 14 days. At day 7, B cell depletion deficiency was tested.

For B-cell transfer model on $ApoE^{-/-}$ mice, 8-week-old female $ApoE^{-/-}$ mice were injected with antibodies against CD19 (75 μg/mouse) and CD20 (50 μg/mouse) to deplete B cells. After 7 days, splenic $CD19^+$ B cells ($2X10^7$ cells/mouse, iv) from $PKM2^{fl/fl}$ or $CD19crePKM2^{fl/fl}$ mice were transferred into B cell-deficient $ApoE^{-/-}$mice, and drinking water containing Hcy (1.8 g/L) was provided for another 28 days.

***Statistics analysis***

ScRNA-seq data were analyzed by R packages Seurat and CellChat. The other data were shown as the mean ± SEM. GraphPad Prism software was used for data analysis. Statistical analysis was performed with one-way or two-way ANOVA followed by Tukey's test for multiple comparisons and with unpaired Student's t test for comparisons between two groups. A value of $P < 0.05$ was considered statistically significant.

**Online Table I. The list of antibodies**

| Antibody | Catalog | Company |
|---|---|---|
| FITC anti-mouse CD45 | 103108 | Biolegend |
| Hoechst 33342 | H3570 | Thermo Fisher Scientific |
| LIVE/DEAD™ Fixable Near-IR Dead Cell Stain Kit | L34975 | Invitrogen |
| CD19 antibody-APC | 152410 | Biolegend |
| B220 antibody-APC/Cyanine7 | 103224 | Biolegend |
| CD4 antibody-APC | 100412 | Biolegend |
| CD4 antibody-APC/Cyanine7 | 100413 | Biolegend |
| CD3 antibody-PE | 100307 | Biolegend |
| F4/80 antibody-APC | 1996380 | eBioscience |
| ICOSL antibody-FITC | MAB1581F | Novus |
| GL7 antibody-PE | 144607 | Biolegend |
| MHC Class II (I-A/I-E) Monoclonal Antibody (M5/114.15.2)-PE | 12-5321-82 | Invitrogen |
| IFN-γ antibody-FITC | 505806 | Biolegend |
| Purified Rat anti-Mouse IA/IE antibody (MHCII) | 556999 | BD |
| CD86 antibody-FITC | 11-0862-82 | Invitrogen |
| DAPI | D1306 | Thermo Fisher Scientific |
| PKM2 (D78A4) XP® Rabbit mAb | 4053S | Cell Signaling Technology |
| Lamin A/C Polyclonal Antibody | A0249 | abclonal |
| β-actin | AC004 | abclonal |
| β-tubulin | AC008 | abclonal |

| | | |
|---|---|---|
| CIITA antibody (H-300) | sc-48797 | Santa Cruz Biotechnology |
| Anti-CREB1 antibody | ab178322 | abcam |
| Recombinant Anti-CREB (phospho S133) antibody | ab32096 | abcam |
| inVivoMab anti-mouse CD19 | BE0150 | bioXcell |
| Rituximab Injection | / | Roche Diagnostics |
| CD11c antibody-FITC | 117305 | Biolegend |
| CD40L antibody-PE | 157003 | Biolegend |

**Online Table II. Primers**

| | | | |
|---|---|---|---|
| qPCR | Cd86 | TCAATGGGACTGCATATCTGCC | GCCAAAATACTACCAGCTCACT |
| | Cd80 | TCAGTTGATGCAGGATACACCA | AAAGACGAATCAGCAGCACAA |
| | Pkm2 | TCGAGAACCATGAAGGCGTC | CGGCGGAGTTCCTCGAATAG |
| | Ciita | AGGCCTATGCCAACATTGCG | CCATAGCATGCTCTTCCGGG |
| | H2-Eb1 | GCGGAGAGTTGAGCCTACG | AGGCCCGTGGACACAATTC |
| | Cd74 | CGCGACCTCATCTCTAACCAT | ACAGGTTTGGCAGATTTCGGA |
| | H2-Aa | AGGTGAAGACGACATTGAGG | AACTCAGGAAGCATCCAGAC |
| | Ciita-pI | ACAGGGACCATGGAGACCATAG | GGGTCGGCATCACTGTTAAGG |
| | Ciita-pIII | GCCGGAGTTGCAAGACCATAG | GGGTCGGCATCACTGTTAAGG |
| | Ciita-pIV | GAGACTGCATGCAGGCAGCAC | GGGTCGGCATCACTGTTAAGG |
| | Il-2 | TGAGCAGGATGGAGAATTACAGG | GTCCAAGTTCATCTTCTAGGCAC |
| | Ifn-γ | ATGAACGCTACACACTGCATC | CCATCCTTTTGCCAGTTCCTC |
| | Cd40lg | CCTTGCTGAACTGTGAGGAGA | CTTCGCTTACAACGTGTGCT |
| | Cd27 | CAGCTTCCCAACTCGACTGTC | GCACCCAGGACGAAGATAAGAA |
| | Cd2 | TTCCTGGGTAGCTTCTTTCTGC | TTGGGGATGTTCAGGGTGATG |
| | β-actin | GTGACGTTGACATCCGTAAAGA | GCCGGACTCATCGTACTCC |
| PCR | Cd19 | GGAGGCAATGTTGTGCTGC | ACAATCACTAGCAAGATGCCC |
| chIP | Ciita-pIII_DNA | CCACAAGGAAGGCAAAGTGC | CTTGTGGGCCAAATTGGGTG |
| | Ciita-pIV_DNA | TCTGGCACCGTGAAGTAACC | TTCCAAGGCAGGTGTTTGGT |
| | Gapdh_DNA | ACCAGGGAGGGCTGCAGTCC | TCAGTTCGGAGCCCACACGC |
| Plasmid | Pkm2-CDS | TACGGCTTCGGTGTGTCAC | TCAAGGTACAGGCACTACACGC |
| | Pkm2-HindIII-F | AAGGATGACAAGCTTATGCCGAAGCCACACAGTGA | |
| | Pkm2-EcoRI-R | TCTATCGATGAATTCTCAAGGTACAGGCACTACACGC | |

## References


Cai J, Deng J, Gu W, Ni Z, Liu Y, Kamra Y, Saxena A, Hu Y, Yuan H, Xiao Q et al (2020) Impact of Local Alloimmunity and Recipient Cells in Transplant Arteriosclerosis. Circ Res 127:974-993

Deng T, Lyon CJ, Minze LJ, Lin J, Zou J, Liu JZ, Ren Y, Yin Z, Hamilton DJ, Reardon PR et al (2013) Class II major histocompatibility complex plays an essential role in obesity-induced adipose inflammation. Cell Metab 17:411-422

Huang da W, Sherman BT, Lempicki RA (2009) Systematic and integrative analysis of large gene lists using DAVID bioinformatics resources. Nat Protoc 4:44-57

Jin S, Guerrero-Juarez CF, Zhang L, Chang I, Ramos R, Kuan CH, Myung P, Plikus MV, Nie Q (2021) Inference and analysis of cell-cell communication using CellChat. Nat Commun 12:1088

Miao Y, Zhao Y, Han L, Ma X, Deng J, Yang J, Lu S, Shao F, Kong W, Wang W et al (2020) NSun2 regulates aneurysm formation by promoting autotaxin expression and T cell recruitment. Cell Mol Life Sci

Oki S, Ohta T, Shioi G, Hatanaka H, Ogasawara O, Okuda Y, Kawaji H, Nakaki R, Sese J, Meno C (2018) ChIP-Atlas: a data-mining suite powered by full integration of public ChIP-seq data. EMBO Rep 19

Stuart T, Butler A, Hoffman P, Hafemeister C, Papalexi E, Mauck WM, 3rd, Hao Y, Stoeckius M, Smibert P, Satija R (2019) Comprehensive Integration of Single-Cell Data. Cell 177:1888-1902 e1821

Winkels H, Ehinger E, Vassallo M, Buscher K, Dinh HQ, Kobiyama K, Hamers AAJ, Cochain C, Vafadarnejad E, Saliba AE et al (2018) Atlas of the Immune Cell Repertoire in Mouse Atherosclerosis Defined by Single-Cell RNA-Sequencing and Mass Cytometry. Circ Res 122:1675-1688

**Supplementary Figure 1. Single live $CD45^+$ cell sorting and quality control of scRNA-seq data.**

**(A)** Gating strategy for sorting single $CD45^+$ (Dead Cell $Stain^-$ $Hoechst^+$) live cells from aortas of $ApoE^{-/-}$, HHcy $ApoE^{-/-}$, and WD $ApoE^{-/-}$ mice for scRNA-seq. (**B)** The concentration of Hcy in plasma of mice. **(C)** Sequencing parameters of 10X Genomics scRNA-seq platform. **(D)** Distribution of gene numbers (nFeature_RNA), UMI (nCount_RNA) and percentage of mitochondrial genes (percent.mt) per cell from raw data. **(E)** Cells within a gene number (nFeature_RNA) of 200-7000, and percent.mt less than 10% were included.

**Supplementary Figure 2. Identification of the types of immune cells.**

**(A)** Top differentially expressed genes among all detected cell clusters. Normalized single-cell gene expression was shown. **(B)** Average expression levels of established immune cell markers in identified cell clusters. **(C-I)**, Violin plots showing the expression levels of indicated marker genes in all identified cell clusters.

**Supplementary Figure 3. Cell cluster identification and functional analyses.**

**(A-F)** Violin plots showing the expression levels of indicated marker genes in all identified cell clusters. **(G-J)** GO enrichment analyses of biological processes in macrophage/monocyte clusters (cluster 1, 2, 4, 11). **(K)** Average expression levels of established cell markers in identified proliferating cell clusters (cluster 9). **(L)** tSNE plots showing color-coded cell clusters of focused analyses on Proliferating cells (cluster 9) in the three groups. **(M)** Bar charts showing the comparison of eight major cell types of Proliferating cells between three groups.

**Supplementary Figure 4. The changes of immune cells in aortas from atherosclerotic mice.**

**(A-D)** Flow cytometry analysis showing the percentages of $B220^+$ cells (B cells), $F4/80^+$ cells (macrophages), and $CD3^+$ cells (T cells) in spleens from mice. * or # $P<0.05$ by one-way ANOVA followed by Tukey's test for multiple comparisons. * indicates the comparison with the $ApoE^{-/-}$ group, # indicates the comparison with the HHcy $ApoE^{-/-}$ group.

**Supplementary Figure 5. Focused analyses on B cells and T cells.**

**(A-D)** Violin plots showing the expression levels of indicated genes among B cell clusters. **(E-H)** Violin plots showing the expression levels of MHCII-related genes. **(I)** Flow cytometry analyses showing the percentages of $CD4^+$ IFN-$\gamma^+$ cells from atherosclerotic aortas. **(J)** Average expression levels of established immune cell markers in identified cell clusters (cluster 6). **(K)** tSNE plots showing color-coded cell clusters of focused analyses on cluster 6 in the three groups. **(L)** Bar charts showing the comparison of five major cell types between three groups. Data represent mean±SEM (n=4 in I). * $P<0.05$ by an unpaired 2-tailed t test (I).

**Supplementary Figure 6. Antigen-presenting B cells are dominant source of MHCII and costimulatory molecule signaling in HHcy-AA.**

**(A)** Circle plots showing the ICOS signaling pathway in the three groups. **(D-I)**, Violin plots showing the expression levels of indicated genes in clusters of antigen-presenting B cells and T cells. **(J-K)** Flow cytometry analyses showing the levels of MHCII and ICOSL in aortic B cells ($B220^+$). **(L-M)** Plasma IL-2 and IFN-γ were measured via ELISA. **(N)** Representative images showing staining of $CD19^+$ cell, $CD4^+$ cells and $GL7^+$ cells in aortas from HHcy $ApoE^{-/-}$ mice. White circles indicated artery tertiary lymphoid organs (ATLOs). Data represent mean±SEM (n=6 in J-K, n=10 in L-M). * $P<0.05$ by one-way ANOVA followed by Tukey's test for multiple comparisons (J-K). * represents the comparisons between the indicated group and the $ApoE^{-/-}$ group. * $P<0.05$ by an unpaired 2-tailed t test (L-M).

**Supplementary Figure 7. Nuclear PKM2 mediated Hcy-induced MHCII expression.**

**(A)** Schematic model of B cell-T cell coculture system. Purified $CD19^+$ splenic B cells from C57BL/6J mice were cultured with or without Hcy (100 μM) for 24 hours, and then cocultured with OT-II mice-derived $CD4^+$ T cells. OVA was added for another 48 hours. **(B-C)** IL-2 and IFN-γ secretion were analyzed via ELISA. **(D-E)** mRNA levels the costimulatory molecules *Cd40, Cd80* and *Cd86* in B cells (D) and *Cd40lg*, *Cd27*, and *Cd2* in T cells (E) were measured via qPCR. **(F-I)** DC-T cell coculture system and macrophage-T cells coculture system were established according to B cell-T cell coculture system. IFN-γ and IL-2 secretion were analyzed via ELISA. **(J)** PKM2 protein levels in purified $CD19^+$ splenic B cells from $PKM2^{fl/fl}$ and $CD19CrePKM2^{fl/fl}$ mice were measured via western blot. **(K)** The DNA and mRNA of Cd19 were detected via PCR. **(L-M)** The protein levels of PKM2 in splenic T cells and peritoneal macrophages isolated from $PKM2^{fl/fl}$ and $CD19cre\text{-}PKM2^{fl/fl}$ mice were measured via western blot. **(N)** Purified $CD19^+$ splenic B cells from $PKM2^{fl/fl}$ and $CD19CrePKM2^{fl/fl}$ mice were cultured with or without Hcy (100 μM) for 24 hours. Representative images showing MHCII staining in B cells. **(O)** Splenic B cells purified from $PKM2^{fl/fl}$ and $CD19CrePKM2^{fl/fl}$ mice (showed as $PKM2^{intact}$ and $PKM2^{null}$ B cells, respectively) were pretreated with or without 100 μM Hcy for 24 hours and then cocultured with OVA-specific $CD4^+$ T cells. OVA was added for another 48 hours. mRNA levels of the costimulatory molecules *Cd40, Cd80* and *Cd86* in B cells were measured via qPCR. **(P)** Violin plots showing the scaled expression scores (Z-scores) of genes related to glycolysis, TCA cycle and pentose phosphate pathway across three groups. **(Q)** Purified $CD19^+$ splenic B cells from C57BL/6J mice were cultured with or without Hcy (100 μM), 2-DG (500 μM), or SKN (0.2 μM) for 24 hours. The expression levels of MHCII in B cells were measured via flow cytometry. **(R)** Purified B cells were cultured with or without TEPP-46 (10 μM) for 24 hours. MHCII expression in B cells were measured via flow cytometry. Data represent the mean±SEM (n=3 in O, n=4 in B-J, Q, n=6 in L-M, n=7-8 in R). * P<0.05 by an unpaired 2-tailed t test (B-J, R). * or # $P<0.05$ by one-way ANOVA followed by Tukey's test for multiple comparisons (O-Q). * indicates the comparison with the Control $PKM2^{intact}$ B cell group (O) or the Control group (Q), # indicates the comparison with the Hcy-treated $PKM2^{intact}$ B cell group (O).

**Supplementary Figure 8. PKM2-induced antigen presentation is critical for B cell-mediated T cell activation.**

**(A)** Schematic plot showing structures of three *Ciita* promoters. **(B)** Schematic plot showing *in vivo* mouse experiments. OT-II mice were injected with antibodies against CD19 and CD20 to deplete B cells. After 7 days, splenic B cells from PKM2$^{fl/fl}$ or CD19crePKM2$^{fl/fl}$ mice were transferred into B cell-deficient OTII-mice, NP-OVA (100 μg) was injected, and drinking water supplemented with Hcy (1.8 g/L) was provided for 14 days. **(C)** B cell depletion efficiency in peripheral blood at day 7 was detected via flow cytometry. **(D)** DCs (CD11c$^+$) and F4/80$^+$ cells in peripheral blood were detected via flow cytometry. **(E)** mRNA levels of MHCII-related genes (*Ciita*, *H2-Eb1*, and *Cd74*) in splenic B cells were measured via qPCR. **(F-G)** IFN-γ and CD40L in splenic CD4$^+$ T cells were analyzed via flow cytometry. **(H-I)** The mRNA levels of costimulatory molecules of B cells and T cells isolated from spleen were measured via qPCR. Data represent the mean±SEM (n=4 in B-E, H-I, n=6-8 in F-G), * *P*<0.05 by an unpaired 2-tailed t test.

**Supplementary Figure 9. The changes of immune cells in HHcy-AA.**

**(A-D)** Flow cytometry analyses showing the percentages of B cells (B220$^+$), T cells (CD3$^+$) and macrophages (F4/80$^+$) in atherosclerotic aortas. **(E-G)** Flow cytometry analyses showing the percentages of CD19$^+$MHCII$^+$ cells (F) and CD4$^+$IFN-γ$^+$ cells (G) in atherosclerotic aortas. Data represent the mean±SEM (n=6 A-G), * *P*<0.05 by an unpaired 2-tailed t test.

# Supplementary Figure 1. Establishment of HHcy mouse model, single live CD45⁺ cell sorting and quality control of scRNA-seq data

A

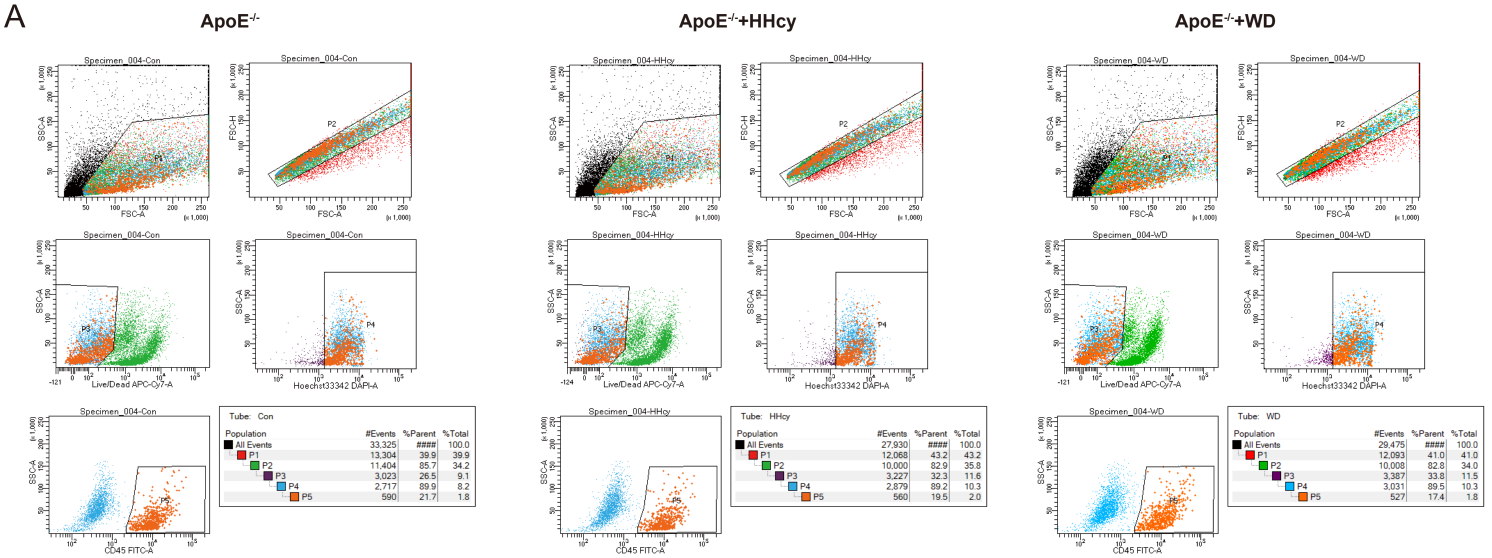


B

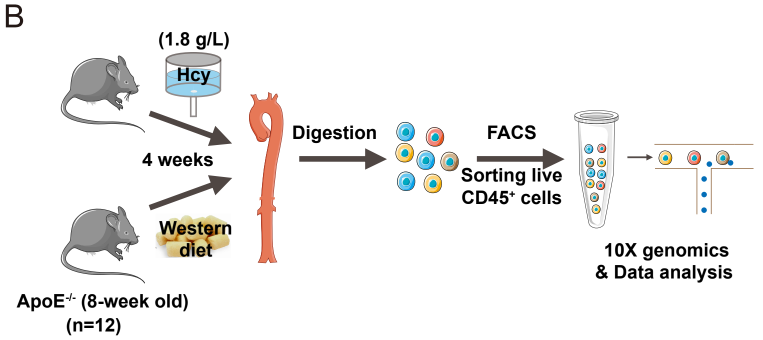


D

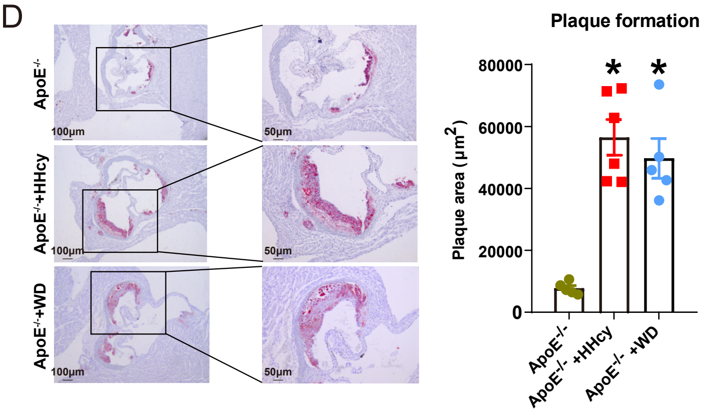


C

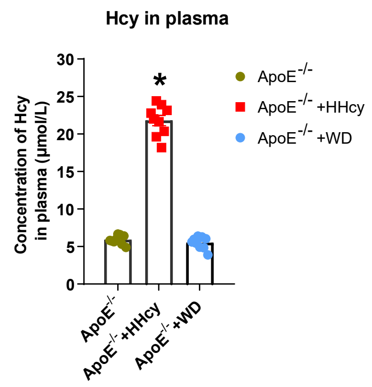


E

| | | $ApoE^{-/-}$ | $ApoE^{-/-}$+HHcy | $ApoE^{-/-}$+WD |
|---|---|---|---|---|
| Sequencing | Number of Reads | 723758443 | 772045620 | 699789550 |
| | Valid Barcodes | 97.3% | 97.3% | 97.1% |
| | Valid UMIs | 99.9% | 99.9% | 99.9% |
| | Sequencing Saturation | 81.2% | 73.0% | 73.6% |
| | Q30 Base in Barcode | 96.2% | 96.3% | 96.2% |
| | Q30 Base in RNA Read | 91.7% | 91.8% | 91.9% |
| | Q30 Base in UMI | 95.6% | 95.7% | 95.7% |
| Cells | Estimated Number of Cells | 7512 | 9753 | 8557 |
| | Fraction Reads in Cells | 90.2% | 91.2% | 90.7% |
| | Mean Reads per Cell | 96347 | 79160 | 81780 |
| | Median Genes per Cell | 2152 | 2134 | 2371 |
| | Total Genes Detected | 20329 | 21424 | 21326 |
| | Median UMI Counts per Cell | 6664 | 7066 | 7678 |
| Mapping | Reads Mapped to Genome | 93.9% | 95.0% | 95.1% |
| | Reads Mapped Confidently to Genome | 91.6% | 92.9% | 94.2% |
| | Reads Mapped Confidently to Intergenic Regions | 4.9% | 4.7% | 5.7% |
| | Reads Mapped Confidently to Intronic Regions | 24.4% | 26.2% | 23.5% |
| | Reads Mapped Confidently to Exonic Regions | 62.3% | 61.9% | 62.7% |
| | Reads Mapped Confidently to Transcriptome | 58.9% | 58.5% | 59.2% |
| | Reads Mapped Antisense to Gene | 1.5% | 1.5% | 1.5% |

F

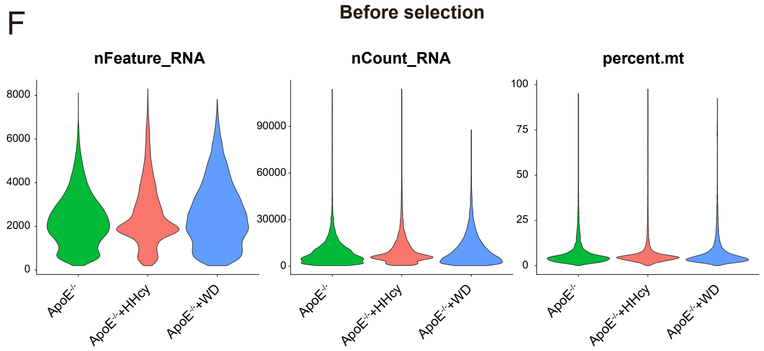


G

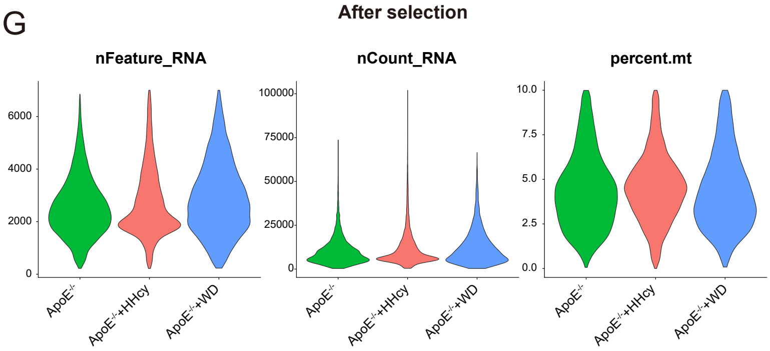

**Supplementary Figure 1. Establishment of HHcy mouse model, single live CD45$^+$ cell sorting and quality control of scRNA-seq data**

**(A)** Gating strategy for sorting single CD45$^+$ (Dead Cell Stain$^-$ Hoechst$^+$) live cells from aortas of ApoE$^{-/-}$, HHcy ApoE$^{-/-}$, and WD ApoE$^{-/-}$ mice for scRNA-seq. **(B)** Workflow showing the establishment of HHcy or western diet (WD)-accelerated atherosclerotic mouse model, aorta digestion, single CD45$^+$ live leukocyte sorting and scRNA-seq. As the control group, ApoE$^{-/-}$ mice (8 weeks old, female) were fed a normal chow diet and normal water for 4 weeks; For HHcy model, ApoE$^{-/-}$ mice were fed a normal chow diet and water with Hcy (1.8 g/L) for 4 weeks; For WD model, ApoE$^{-/-}$ mice were fed a WD and normal water for 4 weeks. **(C)** The concentration of Hcy in plasma of mice. **(D)** Representative images showing cross-sections of aortic roots stained with Oil Red O, to assess and quantify plaque formation and lipid deposition. **(E)** Sequencing parameters of 10X Genomics scRNA-seq platform. **(F)** Distribution of gene numbers (nFeature_RNA), UMI (nCount_RNA) and percentage of mitochondrial genes (percent.mt) per cell from raw data. **(G)** Cells within a gene number (nFeature_RNA) of 200-7000, and percent.mt less than 10% were included.

# Supplementary Figure 2. Identification of the types of immune cells

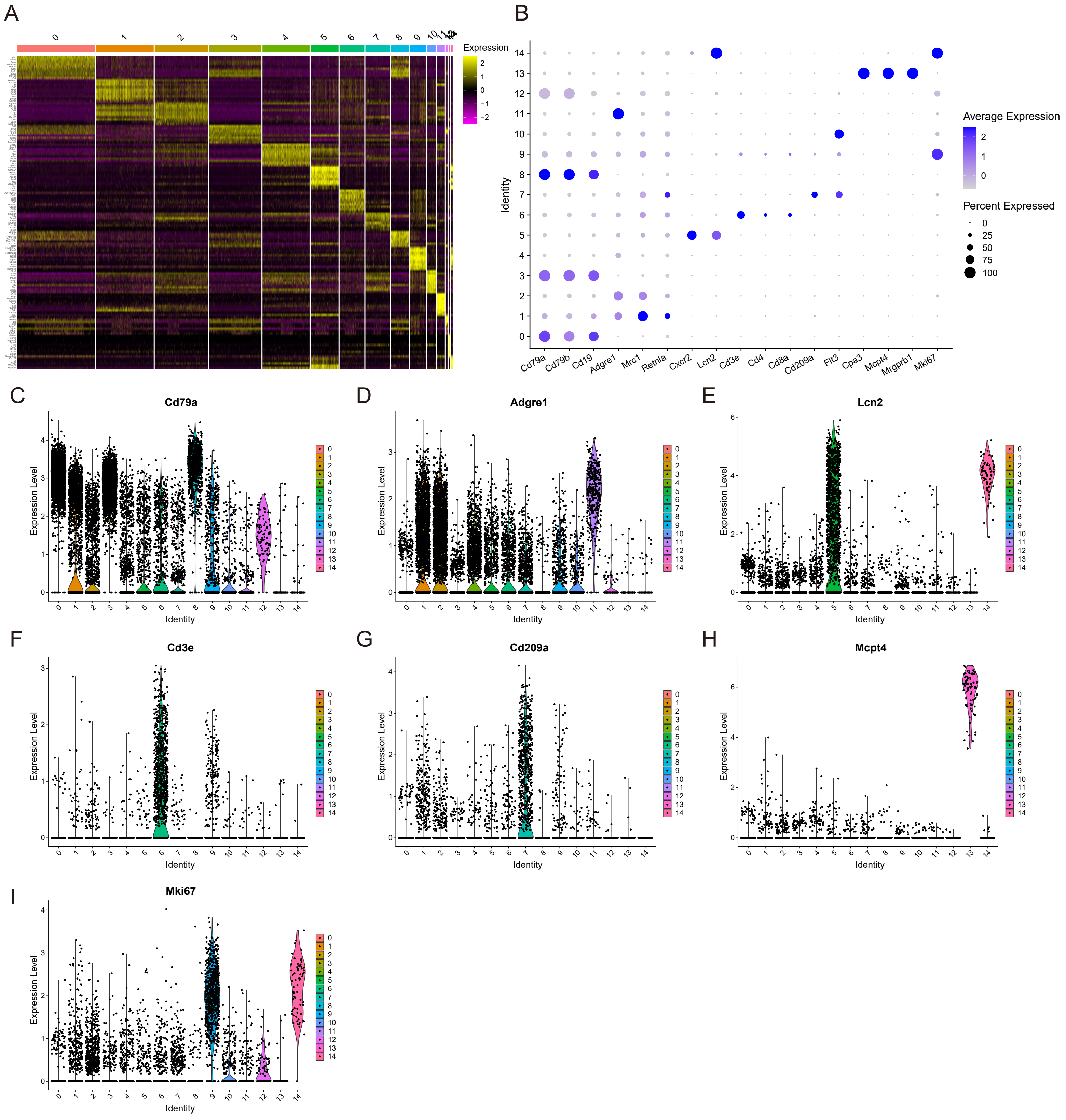

**Supplementary Figure 2. Identification of the types of immune cells.**

**(A)** Top differentially expressed genes among all detected cell clusters. Normalized single-cell gene expression was shown. **(B)** Average expression levels of established immune cell markers in identified cell clusters. **(C-I)**, Violin plots showing the expression levels of indicated marker genes in all identified cell clusters.

# Supplementary Figure 3. Cell cluster identification of B cells and functional analyses of macrophages

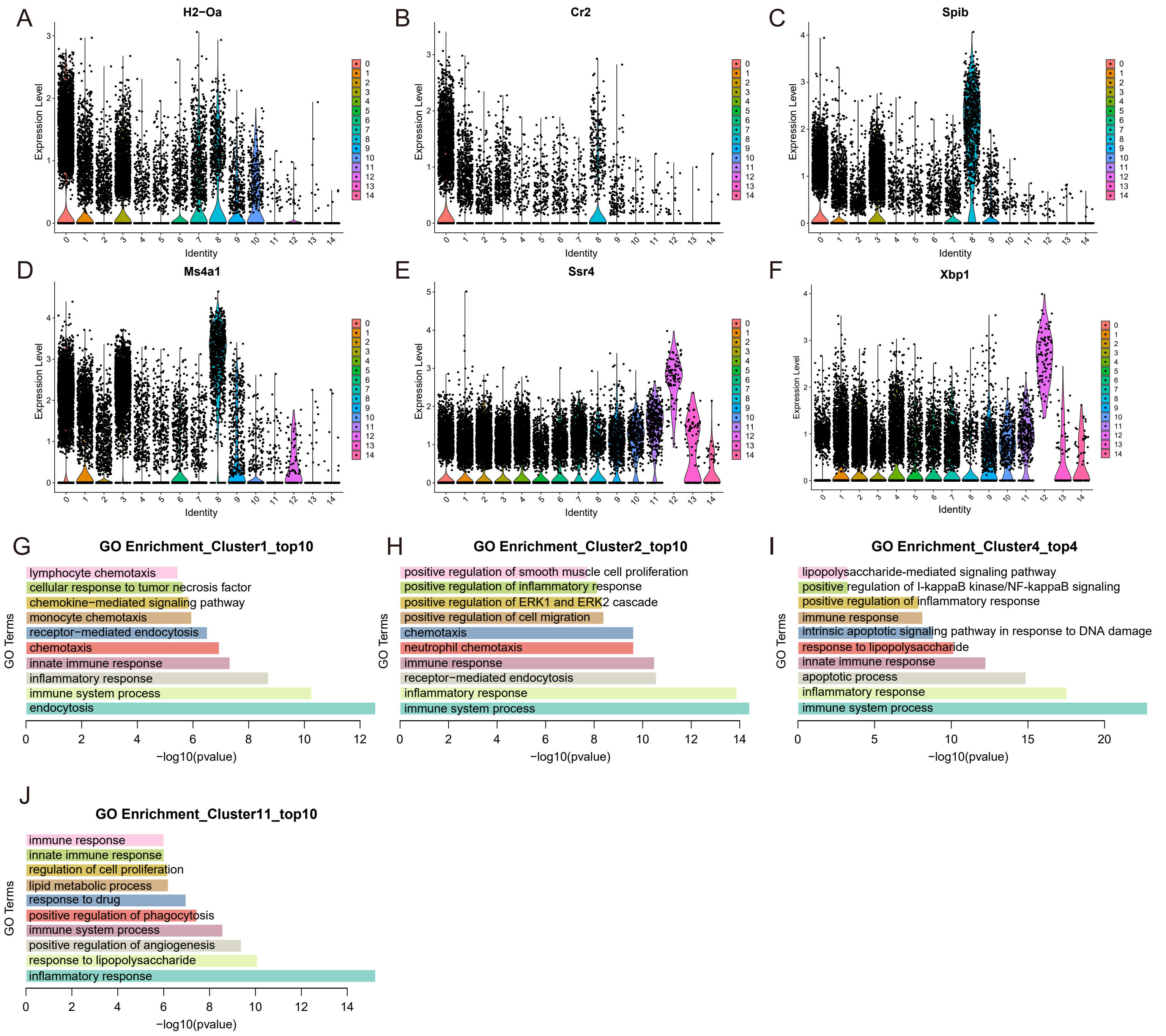

**Supplementary Figure 3. Cell cluster identification of B cells and functional analyses of macrophages.**

**(A-F)** Violin plots showing the expression levels of indicated marker genes in all identified cell clusters. **(G-J)** GO enrichment analyses of biological processes in macrophage/monocyte clusters (cluster 1, 2, 4, 11).

# Supplementary Figure 4. The comparisons of cell clusters and proliferating cell cluster identification

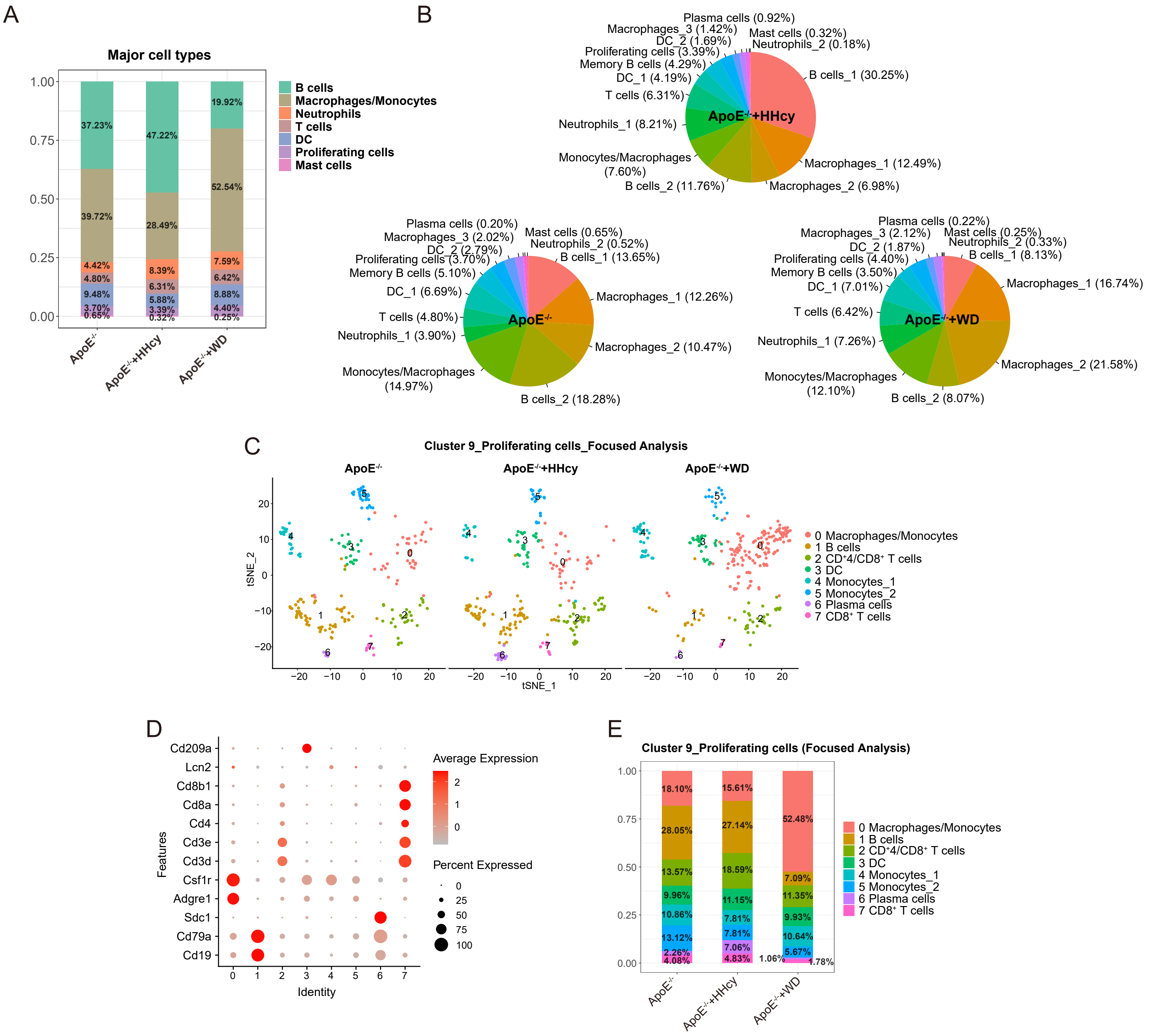

**Supplementary Figure 4. The comparisons of cell clusters and proliferating cell cluster identification.**

**(A)** Bar charts showing the comparison of 6 major cell types between three groups. **(B)** Pie charts showing the percentages of cell clusters in the three groups. **(C)** tSNE plots showing color-coded cell clusters of focused analyses on Proliferating cells (cluster 9) in the three groups. **(D)** Average expression levels of established cell markers in identified proliferating cell clusters (cluster 9). **(E)** Bar charts showing the comparison of eight major cell types of Proliferating cells between three groups.

# Supplementary Figure 5. The changes of immune cells in aortas from atherosclerotic mice

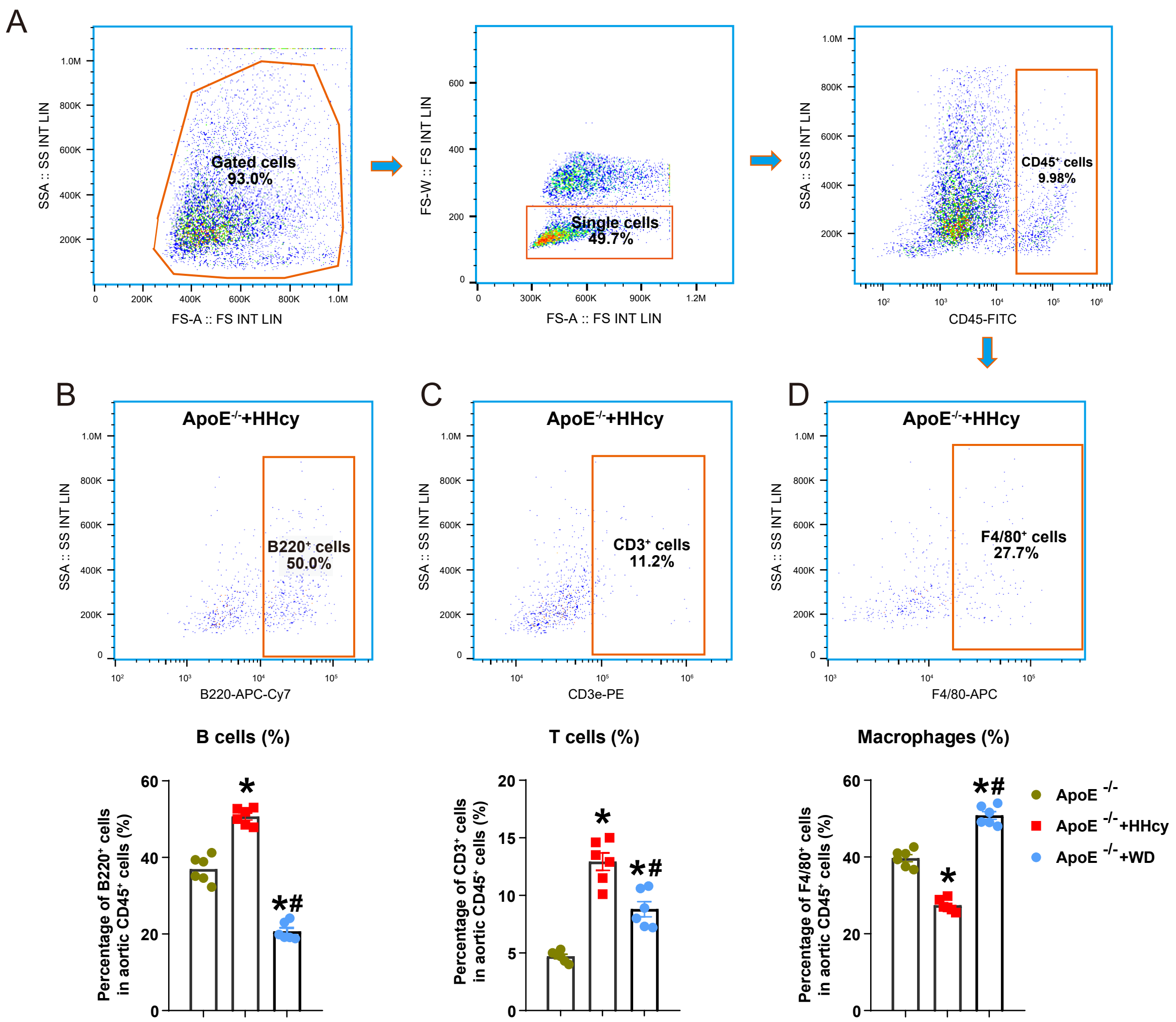

**Supplementary Figure 5. The changes of immune cells in aortas from atherosclerotic mice.** **(A-D)** Flow cytometry analysis showing the percentages of $B220^+$ cells (B cells), $F4/80^+$ cells (macrophages), and $CD3^+$ cells (T cells) in spleens from mice. * or # $P<0.05$ by one-way ANOVA followed by Tukey's test for multiple comparisons. * indicates the comparison with the $ApoE^{-/-}$ group, # indicates the comparison with the HHcy $ApoE^{-/-}$ group.

# Supplementary Figure 6. Focused analyses on B cells and T cells.

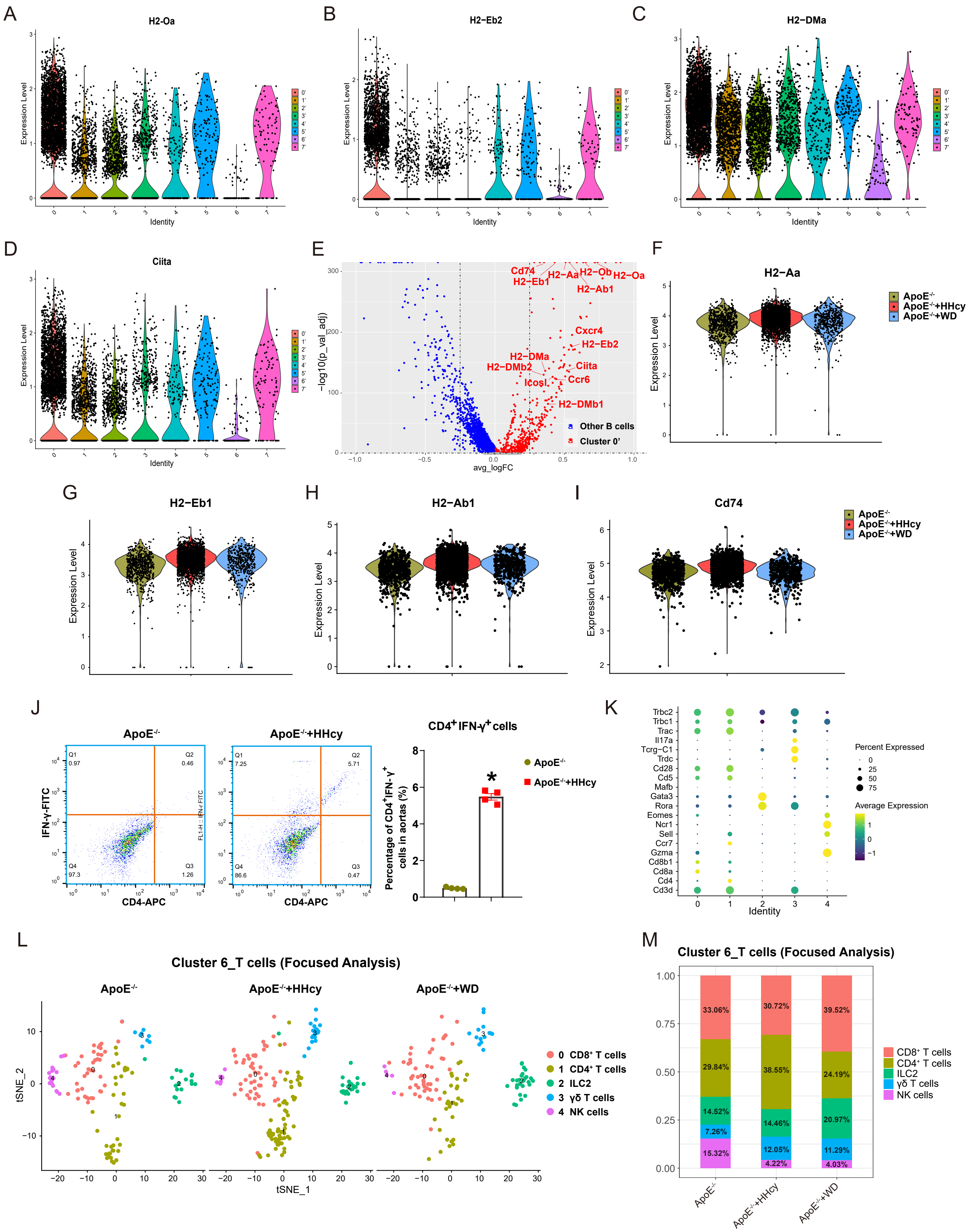

**Supplementary Figure 6. Focused analyses on B cells and T cells.**

**(A-D)** Violin plots showing the expression levels of indicated genes among B cell clusters. **(E)** Volcano plots showing gene features between cluster 0' and the other seven B cells clusters. **(F-I)** Violin plots showing the expression levels of MHCII-related genes. **(J)** Flow cytometry analyses showing the percentages of $CD4^{+}$ IFN-$\gamma^{+}$ cells from atherosclerotic aortas. **(K)** Average expression levels of established immune cell markers in identified cell clusters (cluster 6). **(L)** tSNE plots showing color-coded cell clusters of focused analyses on cluster 6 in the three groups. **(M)** Bar charts showing the comparison of five major cell types between three groups. Data represent mean±SEM (n=4 in J). * $P<0.05$ by an unpaired 2-tailed t test (J).

# Supplementary Figure 7. Antigen-presenting B cells are dominant source of MHCII and costimulatory molecule signaling in HHcy-AA

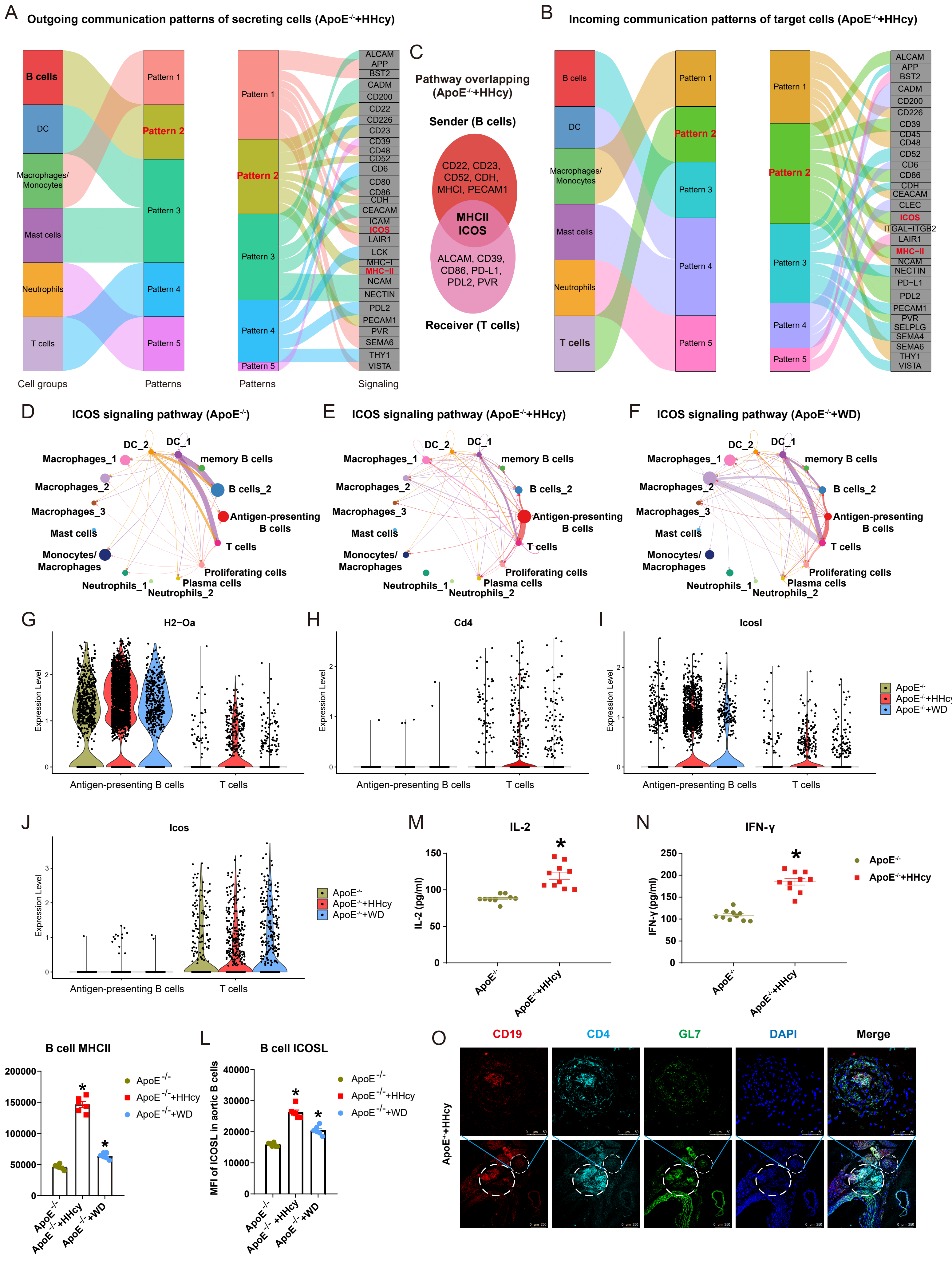

**Supplementary Figure 7. Antigen-presenting B cells are dominant source of MHCII and costimulatory molecule signaling in HHcy-AA.**

**(A)** Alluvial plots showing outgoing communication patterns of cell-cell contact of secreting cells in the HHcy ApoE$^{-/-}$ group. **(B)** Alluvial plots showing incoming communication patterns of cell-cell contact of target cells in the HHcy ApoE$^{-/-}$ group. **(C)** Overlapping pathways between B cells (as senders) and T cells (as receivers) in the HHcy ApoE$^{-/-}$ group. **(D-F)** Circle plots showing the ICOS signaling pathway in the three groups. **(G-J)**, Violin plots showing the expression levels of indicated genes in clusters of antigen-presenting B cells and T cells. **(K-L)** Flow cytometry analyses showing the levels of MHCII and ICOSL in aortic B cells (B220$^{+}$). **(M-N)** Plasma IL-2 and IFN-γ were measured via ELISA. **(O)** Representative images showing staining of CD19$^{+}$ cell, CD4$^{+}$ cells and GL7$^{+}$ cells in aortas from HHcy ApoE$^{-/-}$ mice. White circles indicated artery tertiary lymphoid organs (ATLOs). Data represent mean±SEM (n=6 in K-L, n=10 in M-N). * $P$<0.05 by one-way ANOVA followed by Tukey's test for multiple comparisons (K-L). * represents the comparisons between the indicated group and the ApoE$^{-/-}$ group. * $P$<0.05 by an unpaired 2-tailed t test (M-N).

# Supplementary Figure 8. HHcy enhances the ability of B cell antigen presentation

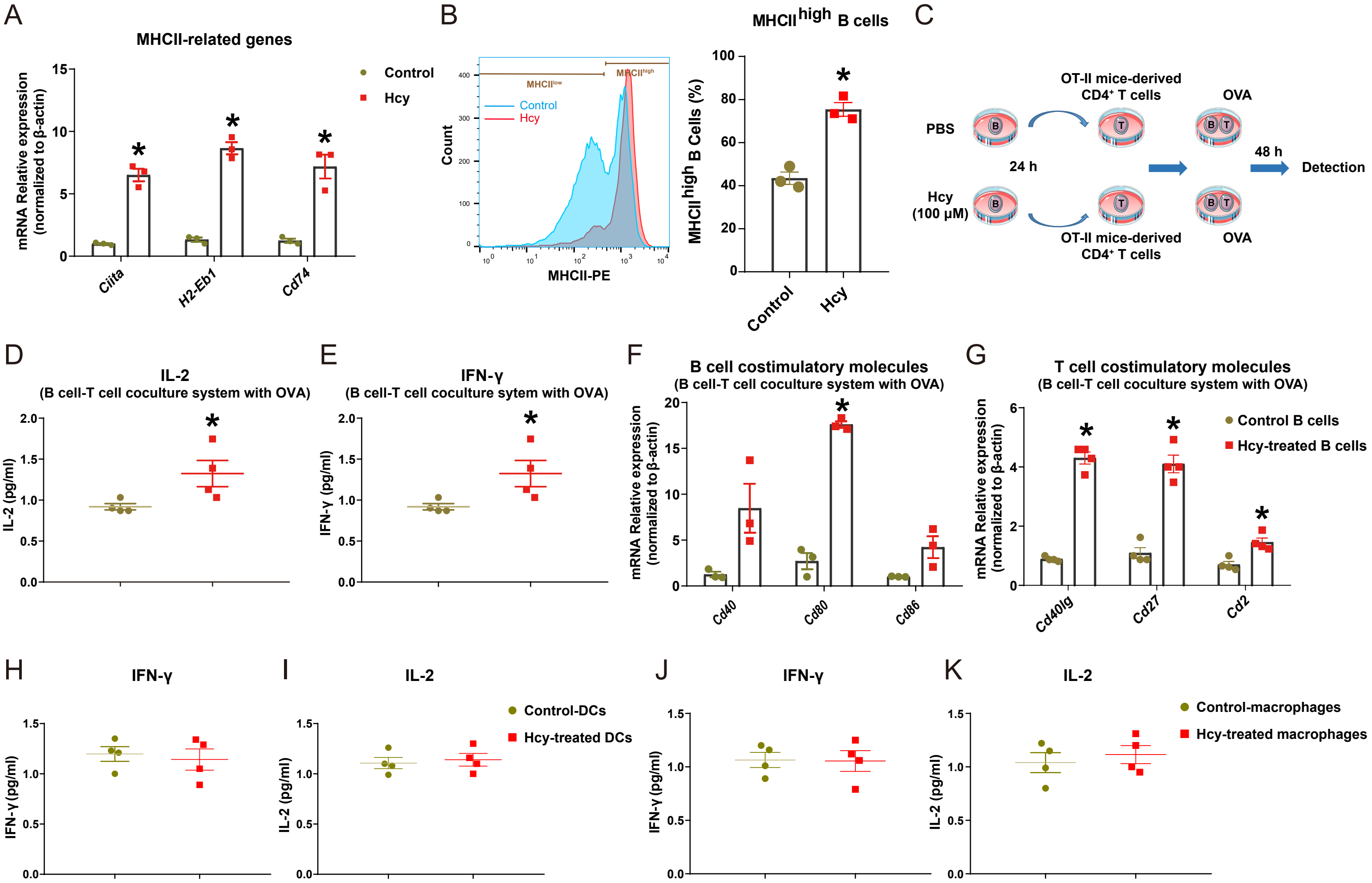

**Supplementary Figure 8. HHcy enhances the ability of B cell antigen presentation.**

**(A-B)** Purified $CD19^+$ splenic B cells from C57BL/6J mice were cultured with or without Hcy (100 μM) for 24 hours. **(A)** mRNA levels of *Ciita*, *H2-Eb1* and *Cd74* in B cells were measured via qPCR. **(B)** The levels of MHCII in B cells were measured and quantified via flow cytometry. **(C)** Schematic model of B cell-T cell coculture system. Purified $CD19^+$ splenic B cells from C57BL/6J mice were cultured with or without Hcy (100 μM) for 24 hours, and then cocultured with OT-II mice-derived $CD4^+$ T cells. OVA was added for another 48 hours. **(D-E)** IL-2 and IFN-γ secretion were analyzed via ELISA. **(F-G)** mRNA levels the costimulatory molecules *Cd40, Cd80* and *Cd86* in B cells (F) and *Cd40lg*, *Cd27*, and *Cd2* in T cells (G) were measured via qPCR. **(H-K)** DC-T cell coculture system and macrophage-T cells coculture system were established according to B cell-T cell coculture system. IFN-γ and IL-2 secretion were analyzed via ELISA.

# Supplementary Figure 9. PKM2-induced antigen presentation is critical for B cell-mediated T cell activation.

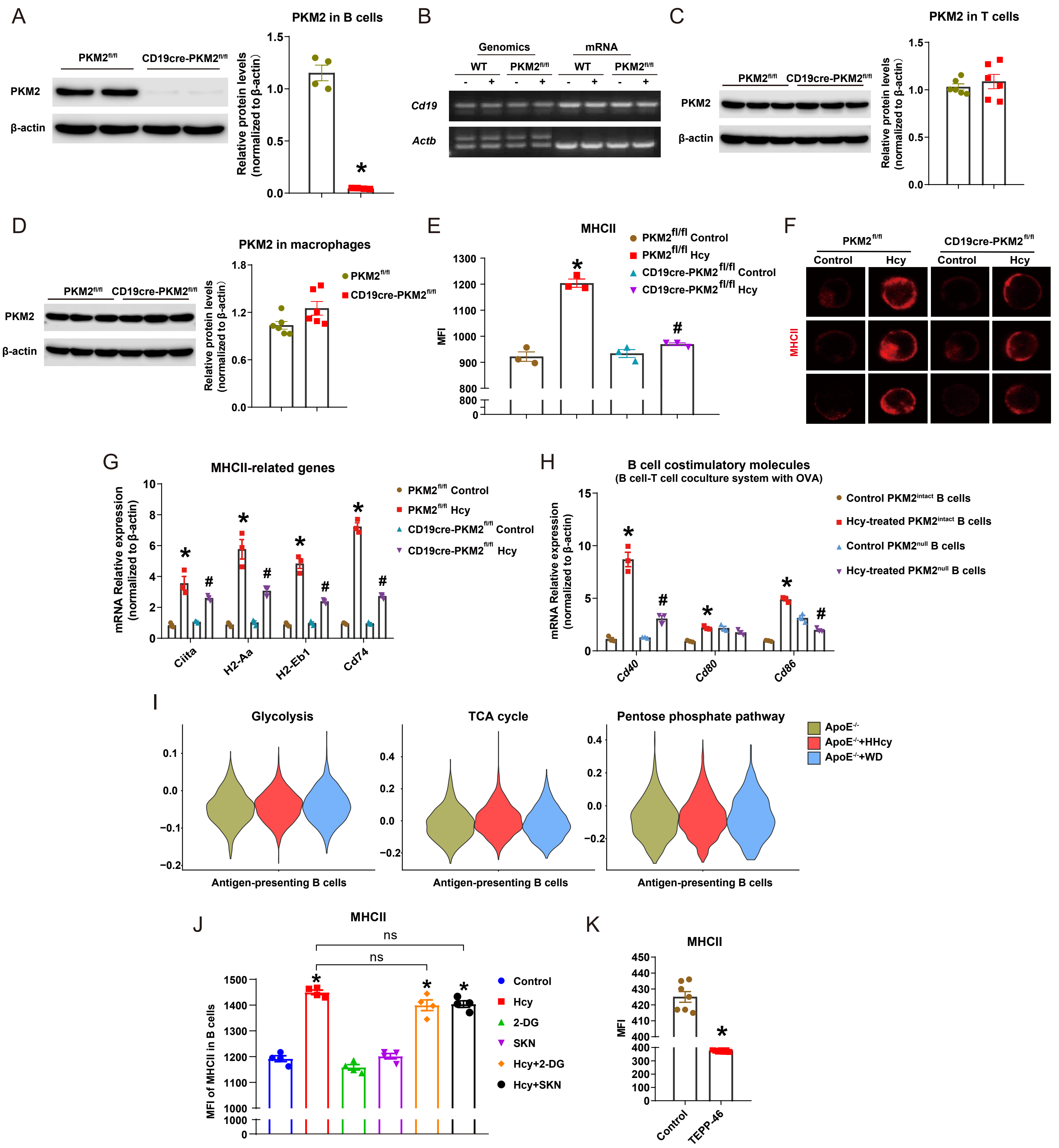

**Supplementary Figure 9. PKM2-induced antigen presentation is critical for B cell-mediated T cell activation.**

**(A)** PKM2 protein levels in purified $CD19^+$ splenic B cells from $PKM2^{fl/fl}$ and $CD19CrePKM2^{fl/fl}$ mice were measured via western blot. **(B)** The DNA and mRNA of Cd19 were detected via PCR. **(C-D)** The protein levels of PKM2 in splenic T cells and peritoneal macrophages isolated from $PKM2^{fl/fl}$ and CD19cre-$PKM2^{fl/fl}$ mice were measured via western blot. **(E-G)** Purified $CD19^+$ splenic B cells from $PKM2^{fl/fl}$ and $CD19CrePKM2^{fl/fl}$ mice were cultured with or without Hcy (100 μM) for 24 hours. **(E)** MHCII expression in B cells was measured via flow cytometry. **(F)** Representative images showing staining of MHCII on B cells. **(G)** mRNA levels of MHCII-related genes (*Ciita*, *H2-Aa*, *H2-Eb1*, and *Cd74)* in B cells were measured via qPCR. **(H)** Splenic B cells purified from $PKM2^{fl/fl}$ and $CD19CrePKM2^{fl/fl}$ mice (showed as $PKM2^{intact}$ and $PKM2^{null}$ B cells, respectively) were pretreated with or without 100 μM Hcy for 24 hours and then cocultured with OVA-specific $CD4^+$ T cells. OVA was added for another 48 hours. mRNA levels of the costimulatory molecules *Cd40, Cd80* and *Cd86* in B cells were measured via qPCR. **(I)** Violin plots showing the scaled expression scores (Z-scores) of genes related to glycolysis, TCA cycle and pentose phosphate pathway across three groups. **(J)** Purified $CD19^+$ splenic B cells from C57BL/6J mice were cultured with or without Hcy (100 μM), 2-DG (500 μM), or SKN (0.2 μM) for 24 hours. The expression levels of MHCII in B cells were measured via flow cytometry. **(K)** Purified B cells were cultured with or without TEPP-46 (10 μM) for 24 hours. MHCII expression in B cells were measured via flow cytometry. Data represent the mean±SEM (n=3 in E, G-H, n=4 in A,J, n=6 in C-D, n=7-8 in K). * $P<0.05$ by an unpaired 2-tailed t test (A, C-D, K). * or # $P<0.05$ by one-way ANOVA followed by Tukey's test for multiple comparisons (E, G-H, J). * indicates the comparison with the $PKM2^{fl/fl}$ Control group (E, G) or the Control $PKM2^{intact}$ B cell group (H), # indicates the comparison with the $PKM2^{fl/fl}$ Hcy group (E, G) or the Hcy-treated $PKM2^{intact}$ B cell group (H).

# Supplementary Figure 10. PKM2 mediated Hcy-induced MHCII expression and T cell activation.

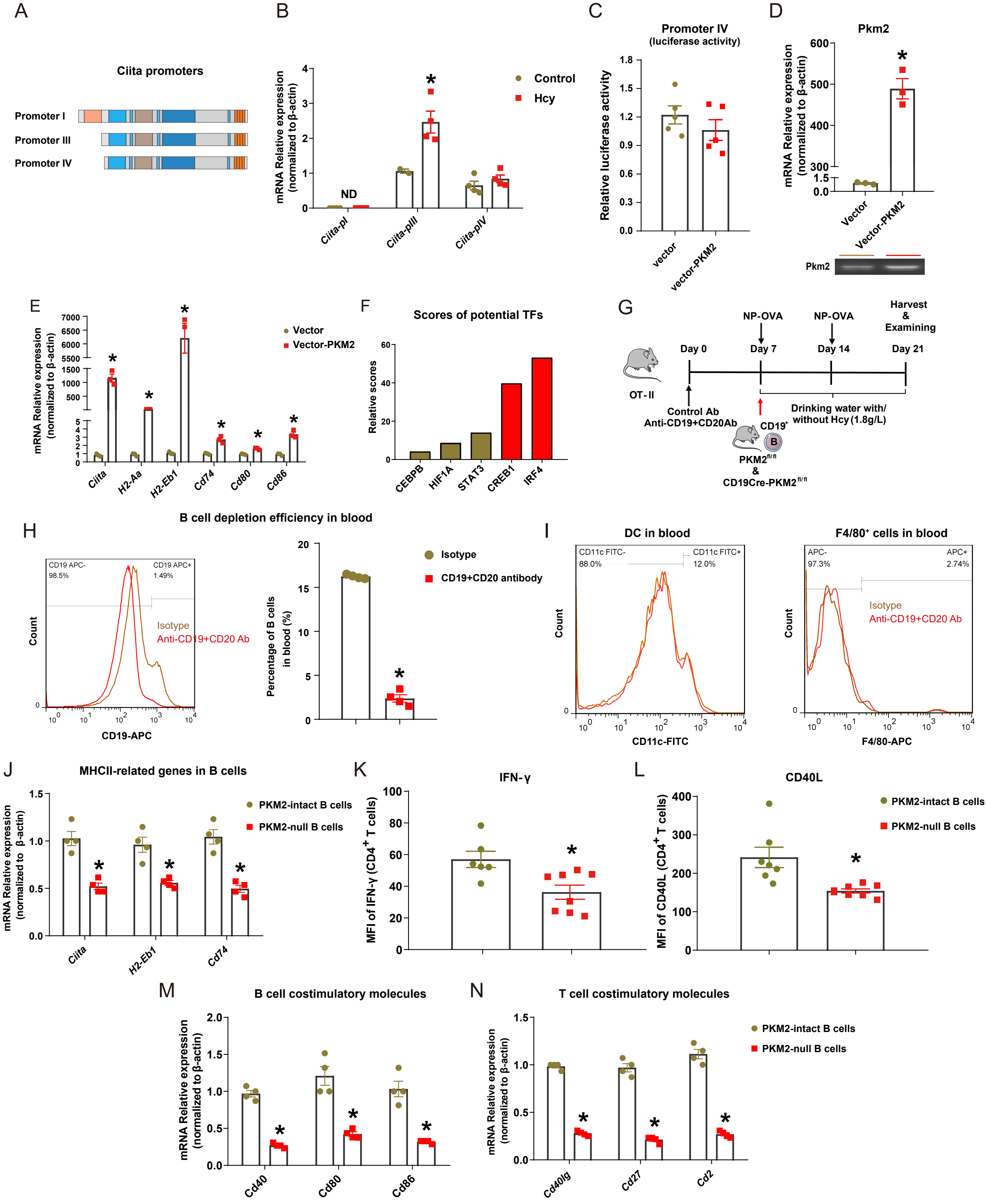

**Supplementary Figure 10. PKM2 mediated Hcy-induced MHCII expression and T cell activation.**

**(A)** Schematic plot showing structures of three *Ciita* promoters. **(B)** Purified $CD19^+$ splenic B cells from C57BL/6J mice were cultured with or without Hcy (100 μM) for 24 hours. The levels of transcripts induced by *Ciita* promoters were measured via qPCR. **(C)** The transcriptional activity of *Ciita* promoter IV with or without PKM2 overexpression was detected via the dual-luciferase system. **(D-E)** Control or *Pkm2* plasmid (1.25 μg/ml, 48 hours) was transfected into primary splenic B cells. mRNA levels of *Pkm2* (D) and MHCII-related genes (E) in B cells were detected via qPCR. **(F)** Relative scores of potential transcription factors (TFs) targeting *Ciita* were predicted using data from ChIP-Atlas. **(G)** Schematic plot showing *in vivo* mouse experiments. OT-II mice were injected with antibodies against CD19 and CD20 to deplete B cells. After 7 days, splenic B cells from $PKM2^{fl/fl}$ or $CD19crePKM2^{fl/fl}$ mice were transferred into B cell-deficient OTII-mice, NP-OVA (100 μg) was injected, and drinking water supplemented with Hcy (1.8 g/L) was provided for 14 days. **(H)** B cell depletion efficiency in peripheral blood at day 7 was detected via flow cytometry. **(I)** DCs ($CD11c^+$) and $F4/80^+$ cells in peripheral blood were detected via flow cytometry. **(J)** mRNA levels of MHCII-related genes (*Ciita*, *H2-Eb1*, and *Cd74*) in splenic B cells were measured via qPCR. **(K-L)** IFN-γ and CD40L in splenic $CD4^+$ T cells were analyzed via flow cytometry. **(M-N)** The mRNA levels of costimulatory molecules of B cells and T cells isolated from spleen were measured via qPCR. Data represent the mean±SEM (n=3 in D-E, n=4 in B, H, J, M-N, n=6-8 in K-L), * $P<0.05$ by an unpaired 2-tailed t test.

# Supplementary Figure 11. The changes of immune cells in HHcy-AA

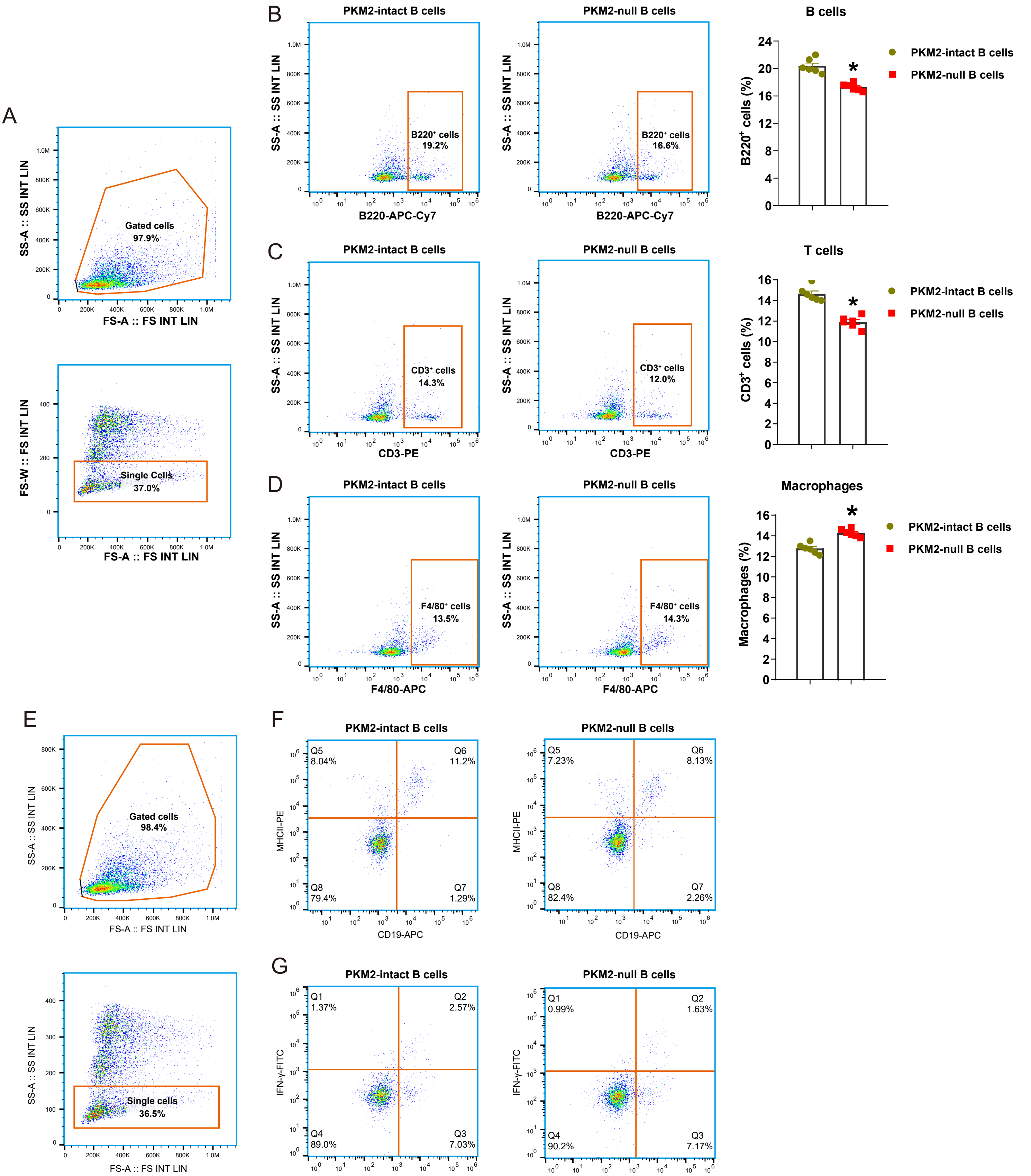

**Supplementary Figure 11. The changes of immune cells in HHcy-AA.**

**(A-D)** Flow cytometry analyses showing the percentages of B cells ($B220^+$), T cells ($CD3^+$) and macrophages ($F4/80^+$) in atherosclerotic aortas. **(E-G)** Flow cytometry analyses showing the percentages of $CD19^+MHCII^+$ cells (F) and $CD4^+IFN\text{-}\gamma^+$ cells (G) in atherosclerotic aortas. Data represent the mean±SEM (n=6 A-G), * $P<0.05$ by an unpaired 2-tailed t test.
